# Measuring the origins of perfectionism with the Roots questionnaire in university students: sociodemographic and educational correlates

**DOI:** 10.3934/publichealth.2026019

**Published:** 2026-03-16

**Authors:** Elena Sandri, Agnese Broccolo, Anna Marchetti, Anna De Benedictis, Giorgia Petrucci, Laura Campanozzi, Mattia Bozzetti, Teresa Rea, Rosaria Alvaro, Maria Grazia De Marinis, Michela Piredda

**Affiliations:** 1 Faculty of Medicine and Health Sciences, Catholic University of Valencia San Vicente Mártir c/Quevedo, 2–46001, Valencia, Spain; 2 Department of Biomedicine and Prevention, University of Rome Tor Vergata, Via Montpellier, 1–00133, Rome, Italy; 3 Department of Medicine and Surgery, Research Unit Nursing Science, Campus Bio-Medico di Roma University, Via Alvaro del Portillo 21–00128 Rome, Italy; 4 Research Unit of Nursing Palliative Care, Fondazione Policlinico Universitario Campus Bio-Medico, Via Alvaro del Portillo, 200–00128, Rome, Italy; 5 Research Unit of Orthopaedic and Trauma Surgery, Fondazione Policlinico Universitario Campus Bio-Medico, Via Alvaro del Portillo, 200–00128 Rome, Italy; 6 Research Unit of Bioethics and Humanities, Campus Bio-Medico di Roma University, Via Alvaro del Portillo, 21–00128 Rome, Italy; 7 Direction of Health Professions, ASST Cremona, 26100 Cremona, Italy; 8 Department Public Health, Università degli Studi di Napoli Federico II, Via Pansini, 5–80131–Naples

**Keywords:** perfectionism, personality, sociodemographic factors, students, university

## Abstract

Perfectionism is increasingly prevalent among university students, yet its developmental antecedents are less understood. This study examined the perceived origins of perfectionism, early family relationships, self-acceptance, and social influences using the 16-item Roots questionnaire (Family, Self, Social) and explored sociodemographic and educational correlates. In a multicenter cross-sectional survey, 2103 Italian students (mean age = 23.4; 76% female) completed the Roots scale. Nonparametric group comparisons and k-means clustering were applied. Students generally reported strong parental support, trust, and autonomy, and moderately positive social acceptance. Vulnerabilities emerged in self-related domains: fear of failure and self-critical tendencies were comparatively higher. Subscale means were as follows: Social = 5.10, Family = 5.06, and Self = 3.75 (1–7 scale). Younger students and those from southern/insular regions reported greater family support. Women presented more fear of failure and self-criticism. Catholics, especially practicing students, reported higher family acceptance/trust alongside greater inadequacy concerns. Health sciences students showed stronger family and self-related resources than peers in science/engineering. Offsite and private-university students displayed more protective indicators; scholarships were associated with heightened internal pressure despite some protective features. No group differences emerged in Social Relationships overall, and total Roots scores did not differ by offsite status or scholarship. Clustering (k = 2) yielded a profile with weaker protective factors and higher fear of failure (consistent with “mixed” perfectionism) and a profile with stronger family/social support, higher self-worth, and lower self-criticism (consistent with “adaptive” perfectionism). Findings suggest that while family cohesion and social trust buffer maladaptive perfectionism, fragile self-perceptions remain central targets. Interventions fostering self-compassion, autonomy-supportive climates, and constructive error management may preserve adaptive striving while mitigating risk.

## 1. Introduction

In recent decades, perfectionism has emerged as an increasingly prevalent phenomenon among younger generations [1]. Longitudinal studies have documented a consistent rise in self-oriented, other-oriented, and socially prescribed perfectionism, with particularly marked increases in younger cohorts compared to previous generations [2]. This trend likely reflects broader sociocultural shifts toward competitiveness, individualism, and meritocracy [3]. Among the groups most affected are university students, who consistently report elevated levels of perfectionism across all dimensions [4].

In this context, university students are increasingly exposed to academic, social, and cultural pressures [5]. They face high performance standards, intense competition, and strict external expectations—from families, educators, and peers—that frame success as the primary measure of personal worth [1],[5]. This climate of expected perfection may encourage the early development of perfectionistic traits, significantly impacting students' psychological and emotional well-being [6].

Perfectionism is commonly defined as a multidimensional personality trait involving the setting of unrealistically high-performance standards, accompanied by overly critical self-evaluations in the event of failure [7],[8]. Two key theoretical models are typically used to interpret this construct: the structural model and the functional model.

The structural model, proposed by Hewitt & Flett [8], conceptualizes perfectionism in three interrelated forms: self-oriented perfectionism (SOP) involves imposing excessively high standards on oneself and harsh self-judgment upon failure, socially prescribed perfectionism (SPP) reflects the belief that one's value depends on meeting the high expectations of others, and other-oriented perfectionism (OOP) entails holding others to unrealistic standards, often accompanied by critical or rigid attitudes. These dimensions are associated with various psychological outcomes [9]–[11]. SPP is strongly linked to psychopathological symptoms, including anxiety, depression, suicidal ideation, and dysfunctional procrastination [12]–[15]. SOP presents a more complex profile, comprising both adaptive components, such as motivation for excellence and achievement orientation [16], and maladaptive aspects, including excessive self-criticism, cognitive rigidity, and vulnerability to disorders such as eating disorders, obsessive beliefs, and chronic worry [11],[12]. The psychological impact of SOP thus appears to depend heavily on individual flexibility and the context in which personal standards are regulated [17].

The functional model distinguishes between adaptive and maladaptive perfectionism. Adaptive perfectionism has been associated with positive outcomes such as lower procrastination, greater self-efficacy, higher self-esteem, better organizational skills, and lower psychological distress [18]–[23]. In contrast, maladaptive perfectionism is characterized by excessive self-criticism, fear of failure, low self-worth, and heightened vulnerability to stress, anxiety, and depression [18],[21]. This dysfunctional profile has been linked to numerous negative consequences on both individual and academic levels, including suicidal ideation, disordered eating, and academic procrastination [24]–[26]. Such traits are also associated with decreased academic satisfaction, burnout [27], declining motivation [28], and, in severe cases, educational dropout. These findings suggest that maladaptive perfectionism constitutes not only a mental health risk but also a substantial obstacle to students' academic and personal fulfilment. It is now widely recognized as a strong predictor of psychological distress [29], especially in the form of performance anxiety and academic burnout [30],[31]. These outcomes are frequently mediated by recurring negative emotions such as fear of failure, guilt, and persistent feelings of inadequacy [32],[33]. Among the perfectionism dimensions, SPP is most consistently associated with psychopathological outcomes, particularly in academic environments where social and academic pressures reinforce the need to meet unrealistic standards to gain approval or success [34]–[36].

Understanding the origins of perfectionism is therefore critical for developing effective prevention and intervention strategies. The Perfectionism Social Disconnection Model (PSDM) posits that perfectionism may stem from early relational experiences marked by insecure attachment, rigid or conditional parental expectations, and a perception of oneself as unworthy of love and approval [37]. These formative experiences may contribute to a self-concept that is highly dependent on external validation. Additionally, pressure from peers and teachers, particularly in competitive and performance-focused environments, can reinforce maladaptive patterns of self-evaluation and emotional regulation [3],[38].

Despite growing interest in the developmental roots of perfectionism, most existing assessment tools focus primarily on its behavioral and cognitive manifestations, often neglecting the relational and affective dynamics underlying its emergence [39]. The Roots questionnaire, recently developed and validated in Italy, aims to systematically evaluate the perceived etiological factors of perfectionism among university students [40]. It examines students' perceptions of early relational experiences, self-worth, fear of failure, and social dynamics, offering a more comprehensive understanding of how perfectionism develops.

The present study aims to investigate the perceived origins of perfectionism, understood as early relational experiences, self-acceptance, and social influences, in university students using the Roots questionnaire. Specifically, it explores how these origins relate to the sample's sociodemographic and educational characteristics. By analyzing students' perceptions of family relationships, self-image, and social expectations, the study seeks to identify patterns associated with variables such as age, gender, and academic field. Ultimately, the goal is to enhance our understanding of the relational and psychological factors that foster perfectionism in academic settings, with a view toward preventing psychological distress and promoting student well-being.

## 2. Materials and methods

### 2.1. Study design

This was a multicenter cross-sectional observational study.

### 2.2. Instrument

The origins of perfectionism were measured using the 16-item Roots of Perfectionism scale (Roots) validated by Piredda et al. [40]. This scale assesses three factors: Relationships with family (Family), measured by seven items (#1, #3, #4, #6, #8, #11, #13), Relationships with the self (Self), measured by six items (#2, #5, #9, #12, #14, #15), and Social relationships (Social), measured by three items (#7, #10, #16). In the validation study, the structure validity of the scale was confirmed as a three-factor model and a second-order factor through confirmatory factor analysis (CFA) with the following fit indices: χ^2^(99, n = 469) = 228.618, p < 0.0001; RMSEA = 0.053 (IC 90%, 0.044–0.062), p = 0.291; CFI = 0.952; TLI = 0.941; SRMR = 0.050. Construct validity supported the expected relationships between scores of the Roots scale and subscales and indicators of perfectionism and perceived stress. Regarding reliability, omega coefficients for Family, Self, and Social were 0.89, 0.86, and 0.67, respectively, while the Global Reliability index and the OmegaH for the second-order factor were 0.74 and 0.73, respectively. The intraclass correlation coefficient for Roots was 0.71 (95% CI: 0.58–0.81, p < 0.001), showing moderate test–retest stability [40].

**Table 1.** Items of the Roots scale.

Items of Roots
Roots1. I perceived myself as receiving love from my parents.
Roots2. When I make a mistake, I believe that I am at fault.
Roots3. I feel accepted by my parents as I am.
Roots4. I believe that my opinion is important to my family.
Roots5. No matter what I do, I feel inadequate.
Roots6. I trust my parents.
Roots7. I feel accepted by others as I am.
Roots8. I believe that my parents trust me.
Roots9. I am afraid of failing.
Roots10. I feel trusted by others.
Roots11. My parents have given me the freedom to choose in important matters.
Roots12. I believe I am worthy as a person, regardless of my mistakes.
Roots13. My parents are satisfied with my achievements based on my efforts.
Roots14. When I make a mistake, I feel like a failure.
Roots15. I feel like I don't have much to be proud of.
Roots16. I feel I can trust others.

Items are rated on a 7-point Likert scale from 1 (strongly disagree) to 7 (strongly agree) (see Table 1). The overall Roots score is calculated by summing all the items, with a range from 16 to 112. The scores of the items reflecting negative statements (#2, #5, #9, and #14) are reversed so that higher Roots scores mean perceptions and beliefs that are protective toward maladaptive perfectionism [40].

Data on the sample socio-demographic and academic information, including sex, age, religion and religious practice, study area and level, type of university (public or private), offsite study status, and scholarship or College of Merit placement, was also gathered. Age was classified in three groups: young (18–24 years), young adults (25–39 years), and middle-aged (40–62 years).

### 2.3. Data analysis

The dataset was first preprocessed to exclude invalid, inconsistent, or extreme entries, such as inaccurate responses and statistical outliers. Following data cleaning, distributional assumptions were evaluated. Given the large sample size, normality was primarily assessed using skewness and kurtosis indices, as formal normality tests are known to be overly sensitive in large samples. Six variables showed skewness or kurtosis values exceeding |1|, indicating departures from normality (see Table 2). Visual inspection of Q–Q plots further supported these findings [41].

In light of the observed non-normal distributions, nonparametric statistical procedures were employed as appropriate: the Chi-square test for categorical variables, and the Mann–Whitney U or Kruskal–Wallis tests for comparisons between two or more groups. For post-hoc analyses following multiple-group comparisons, the Dwass–Steel–Critchlow–Fligner (DSCF) pairwise comparison test was used [42]. Given the large number of univariable comparisons, we controlled for multiple testing using the Benjamini–Hochberg false discovery rate (FDR) procedure at q = 0.05. The adjustment was applied within each family of related tests corresponding to the sets of group-comparison analyses reported in the same table/panel. Post-hoc pairwise comparisons following Kruskal–Wallis were performed using the DSCF procedure (family-wise control within the pairwise set), whereas pairwise Wilcoxon tests (when used) were adjusted using Holm's method [42]. Confounder-adjusted multivariable regression models were prespecified and are reported with unadjusted coefficient-level p-values.

Sociodemographic categorical variables are reported as counts and percentages, whereas continuous variables are summarized as means with standard deviations.

The instrument's reliability in this sample was assessed by computing the McDonald's Omega coefficient for each factor [43] and the Global Reliability index [44] and the OmegaH [45] for the whole scale.

Additionally, to enhance the interpretation of statistical significance in such a large sample, the effect size r (rank-biserial correlation coefficient) for the Mann–Whitney U test or epsilon-squared (ε²) for Kruskal–Wallis were incorporated. These measures provided a more nuanced view of the relationships between variables. Specifically, an r value or ε² less than 0.3 indicates a small effect, while values above this threshold suggest a larger effect.

**Table 2.** Roots questionnaire scores for the whole sample (N = 2103 university students).

	M	SD	M^	SD^	Skewness	Kurtosis	ITC	Alpha	Global *ω*	*ωH*
Relationships with family	35.40	7.06	5.06^	1.01^				0.74		
1. I perceived myself as receiving love from my parents	5.94	1.42			−1.472	1.768	0.522			
3. I feel accepted by my parents as I am	5.52	1.65			−1.052	0.251	0.611			
4. I believe that my opinion is important to my family	4.12	1.92			−0.029	−1.095	0.215			
6. I trust my parents	4.41	1.91			−0.158	−1.107	0.450			
8. I believe that my parents trust me	5.76	1.42			−1.282	1.375	0.625			
11. My parents have given me the freedom to choose	5.83	1.40			−1.336	1.397	0.426			
13. My parents are satisfied with my achievements	3.85	1.78			0.155	−0.870	0.266			
Relationships with the self	22.50	6.89	3.75^	1.15^				0.76		
2. When I make a mistake, I believe that I am at fault§	3.18	1.71			0.589	−0.407	0.572			
5. No matter what I do, I feel inadequate§	4.05	1.80			−0.003	−1.020	0.356			
9. I am afraid of failing§	2.33	1.60			1.228	0.863	0.562			
12. I believe I am worthy as a person	5.77	1.36			−1.284	1.620	0.391			
14. When I make a mistake, I feel like a failure§	3.44	1.89			0.392	−0.875	0.690			
15. I feel like I don't have much to be proud of	3.74	1.80			0.182	−0.859	0.467			
Social relationships	15.30	3.37	5.10^	1.12^				0.35		
7. I feel accepted by others as I am	5.45	2.04			−1.167	−0.015	0.096			
10. I feel trusted by others	5.31	1.19			−0.745	1.014	0.244			
16. I feel I can trust others	4.50	1.87			−0.267	1.028	0.217			
Roots	73.20	14.30	4.58^	0.89^					0.88	0.67

Note: § = indicates an item that has been reverse-scored to ensure that higher values consistently represent greater protection; M = mean; SD = standard deviation; ^ = weighted value for the number of items; ω = Omega coefficient; ωH = Omega hierarchical coefficient.

**Table 3.** Multiple linear regression models between Relationships with Family and sociodemographic factors.

Relationships with family (R = 0.266; R^2^ = 0.0709)					
Predictor	β	SE	95% CI	t	p
Constant^a^	36.9773	1.02	[34.98, 38.98]	36.2402	<0.001*
Sex
Female–male	−1.6718	0.358	[−2.37, −0.97]	−4.6691	<0.001*
Age
Young adults–young	−0.3179	0.387	[−1.08, 0.44]	−0.8216	0.411
Middle age–young	0.8136	0.888	[−0.93, 2.55]	0.9165	0.359
Religious
Other religion–catholic	−2.2921	0.631	[−3.53, −1.06]	−3.6304	<0.001*
Atheist/Agnostic–catholic	−2.7018	0.363	[−3.41, −1.99]	−7.4369	<0.001*
Indifferent–catholic	−1.494	0.458	[−2.39, −0.60]	−3.263	<0.001*
Area of study
Science–health science	−0.1703	0.71	[−1.56, 1.22]	−0.2399	0.810
Humanities–health science	−0.1208	0.553	[−1.20, 0.96]	−0.2183	0.827
Engineering–health science	−0.9919	0.601	[−2.17, 0.19]	−1.6511	0.099
Others–health science	0.9406	0.968	[−0.96, 2.84]	0.9718	0.331
Level of study
Master's degree–Bachelor's degree	0.7724	0.429	[−0.07, 1.61]	1.7994	0.072
Location
Center–North	−0.4595	0.534	[−1.51, 0.59]	−0.86	0.390
South–North	0.7182	0.766	[−0.78, 2.22]	0.9381	0.348
Islands–North	1.1887	0.514	[0.18, 2.20]	2.3109	0.021*
Private University
Yes–No	1.9482	0.834	[0.31, 3.58]	2.3349	0.020*

Note: ^a^ = Level of reference. SE = Standard errors. CI = confidence intervals (computed as β ± 1.96 × SE).

To reduce the risk of confounding, multiple linear regression models were conducted in which each questionnaire subscale was used as the dependent variable. The models were adjusted for sex, age, region, field of study, and type of institution (public/private) (Tables 3–5).

**Figure 1.**
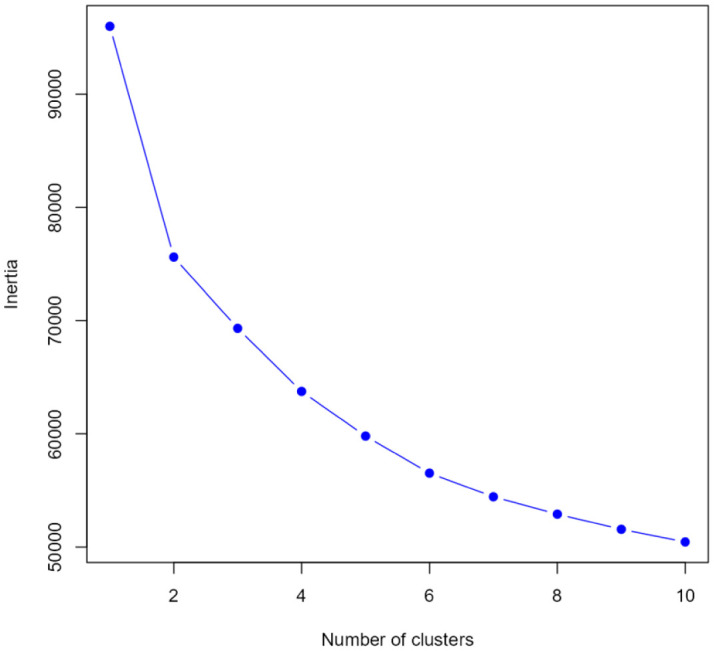
Identification of the elbow point, the method used to determine the optimal number of clusters.

To further explore behavioral patterns and latent structures within the dataset, clustering methods were applied to identify groups of individuals sharing similar characteristics. Clustering provides an effective means of organizing data into meaningful subgroups, in which members exhibit greater similarity to each other than to those in different clusters. This approach helps to uncover hidden structures and natural partitions within complex datasets. The k-means clustering algorithm was selected as the primary method, owing to its suitability for partitioning data into compact, internally consistent clusters. Among its implementations, the Hartigan–Wong algorithm was employed for its demonstrated effectiveness in minimizing within-cluster sum of squares, thereby producing stable and well-separated groupings. In addition, the k-medoids algorithm was considered for its robustness to outliers and ability to mitigate the influence of noise—unwanted variability or random error that can obscure underlying patterns [46]. The optimal number of clusters was determined using the elbow method [47], which involves plotting the total within-cluster sum of squares against increasing values of k. The elbow plots (Figure 1) displayed a distinct inflection at k = 2, indicating that a two-cluster solution offered the most parsimonious and interpretable structure across both dimensions. Beyond this point, additional clusters yielded minimal improvements in model fit, further supporting the selection of a two-group configuration.

**Table 4.** Multiple linear regression models between Relationships with the Self and sociodemographic factors.

Relationships with the self (R = 0.305; R^2^ = 0.0927)					
Predictor	Β	SE	95% CI	t	p
Constant^a^	24.6082	0.514	[23.60, 25.62]	47.889	<0.001*
Sex
Female–male	−2.9074	0.345	[−3.58, −2.23]	−8.427	<0.001*
Age
Young adults–young	1.241	0.371	[0.51, 1.97]	3.344	<0.001*
Middle age–young	6.1221	0.855	[4.45, 7.80]	7.163	<0.001*
Religious
Other religion–catholic	−0.1196	0.607	[−1.31, 1.07]	−0.197	0.844
Atheist/Agnostic–catholic	−1.5874	0.35	[−2.27, −0.90]	−4.54	<0.001*
Indifferent–catholic	−0.4338	0.441	[−1.30, 0.43]	−0.983	0.326
Area of study
Science–health science	0.0857	0.684	[−1.25, 1.43]	0.125	0.900
Humanities–health science	0.3674	0.529	[−0.67, 1.40]	0.694	0.488
Engineering–health science	−2.1547	0.579	[−3.29, −1.02]	−3.723	<0.001*
Others–health science	1.3036	0.932	[−0.52, 3.13]	1.398	0.162
Level of study
Master's degree–Bachelor's degree	0.5687	0.413	[−0.24, 1.38]	1.377	0.169
Location
Center–North	−0.701	0.512	[−1.70, 0.30]	−1.37	0.171
South–North	0.3062	0.679	[−1.02, 1.64]	0.451	0.652
Islands–North	1.2276	0.485	[0.28, 2.18]	2.532	0.011*
Private University
Yes–No	1.345	0.463	[0.44, 2.25]	2.903	0.004*

Note: ^a^ = Level of reference. SE = Standard errors. CI = confidence intervals (computed as β ± 1.96 × SE).

**Table 5.** Multiple linear regression models between social relationships and sociodemographic factors.

Social relationships (R = 0.454; R^2^ = 0.206)					
Predictor	β	SE	95% CI	t	p
Constant^a^	15.6835	0.235	[15.22, 16.14]	66.691	<0.001*
Sex
Female–male	0.0962	0.158	[−0.21, 0.41]	0.609	0.542
Age
Young adults–young	0.1224	0.17	[−0.21, 0.46]	0.721	0.471
Middle age–young	1.2039	0.391	[0.44, 1.97]	3.078	0.002*
Religious
Other religion–catholic	−0.2593	0.278	[−0.80, 0.29]	−0.933	0.351
Atheist/Agnostic–catholic	−0.5742	0.16	[−0.89, −0.26]	−3.588	<0.001*
Indifferent–catholic	−0.4012	0.202	[−0.80, −0.01]	−1.986	0.047*
Area of study
Science–health science	−0.0934	0.313	[−0.71, 0.52]	−0.299	0.765
Humanities–health science	0.3446	0.242	[−0.13, 0.82]	1.423	0.155
Engineering–health science	−0.6969	0.265	[−1.22, −0.18]	−2.631	0.009*
Others–health science	0.0936	0.427	[−0.74, 0.93]	0.219	0.826
Level of study
Master's degree–Bachelor's degree	0.3216	0.189	[−0.05, 0.69]	1.702	0.089
Location
Center–North	−0.0685	0.234	[−0.53, 0.39]	−0.292	0.770
South–North	2.0794	0.311	[1.47, 2.69]	6.692	<0.001*
Islands–North	0.7098	0.222	[0.27, 1.14]	3.199	0.001*
Private University
Yes–No	−3.5591	0.212	[−3.97, −3.14]	−16.784	<0.001*

Note: ^a^ = Level of reference. SE = Standard errors. CI = confidence intervals (computed as β ± 1.96 × SE).

Finally, a latent profile analysis (LPA) was conducted on the n = 16 R items. LPA is a person-centered statistical technique that identifies unobserved subgroups within a population based on individuals' response patterns, allowing researchers to detect qualitatively distinct profiles rather than assuming a single homogeneous distribution. Models with one to six profiles were estimated using Gaussian mixture modeling with varying within-profile variances and zero covariances (model 2 in tidyLPA, implemented via the mclust package) [48]. Solutions with five and six profiles failed to converge reliably and were therefore not considered further. Fit indices for the 1–4 profile solutions are reported in Table 6. Both Aikaike Information Criterion (AIC) and Bayesian Information Criterion (BIC) decreased monotonically as the number of profiles increased, with the 4-profile solution showing the best balance between fit and parsimony (AIC = 83,594.90; BIC = 84,335.20). Entropy values were high for all multi-profile solutions and increased from 0.88 for the 2-profile model to 0.93 for the 4-profile model, indicating good classification precision. An analytic hierarchy process combining the AIC, BIC, approximate weight of evidence (AWE), classification likelihood criterion (CLC), and Kullback information criterion (KIC) similarly identified the 4-profile solution as optimal [49]–[53].

Statistical significance was defined at p < 0.05. Statistical analyses were performed using Jamovi (Version 2.6.28) [54], while LPA analysis was conducted using R 4.5.0 [55]. Cluster plots were generated in Jamovi, with supplementary visualizations and figure refinements completed in Microsoft Excel.

**Table 6.** Fit indices for latent profile models with 1–4 profiles.

Model	Number of profiles	LogLik	AIC	BIC	Entropy	Smallest class proportion (*n*_min_)	Largest class proportion (*n*_max_)
2	1	−47,736.0	95,536.88	95,717.72	1.00¹	1.00¹	1.00¹
2	2	−43,630.0	87,389.83	87,757.15	0.88	0.49	0.51
2	3	−42,344.0	84,884.45	85,438.26	0.90	0.21	0.40
2	4	−41,666.0	83,594.90	84,335.20	0.93	0.11	0.37

Note: ¹ = For the 1-profile solution, entropy and class proportions are trivial and not substantively interpretable.

### 2.4. Sample and data collection

The sample included undergraduate and master's students attending Italian universities. Students aged less than 18 years were excluded. Convenience sampling was conducted through institutional mailing lists of several Italian universities and personal contacts of the investigators. Data were collected between January 2024 and March 2025 through a multicenter dissemination strategy involving several universities in northern, central, and southern Italy, including the islands. The study followed the STROBE guidelines for observational research [56].

### 2.5. Ethical approval of research

The study was conducted in accordance with the principles of the Helsinki Declaration [57]. The relevant Ethics Committees approved the study prior to commencement [Protocol FPUCBM 001.23(45.22) OSS19 April 2023 and 75.23CET2 cbm, 26 October 2023]. Data protection and confidentiality of participant identities were guaranteed in compliance with current data protection regulations. Participants' informed consent to study participation and data handling was provided online before filling in and returning the questionnaire.

## 3. Results

### 3.1. Sample description

Table 7 reports the socio-demographic profile of the participants. In total, 2103 university students took part in the study, with a higher proportion of females (76.2%) compared to males (23.8%). The mean age was 23.4 years (SD = 5.68). Most students were aged 18–24 years (76.46%), followed by those aged 25–39 years (20.45%), while only a small fraction were in the 40–62 age range (3.09%).

Regarding religious affiliation, over half of the sample identified as Catholic (52.4%). Atheists/agnostics accounted for 27.2%, while 13.9% described themselves as indifferent. Smaller groups identified as Orthodox (1.9%), members of other Christian denominations (1.9%), Muslim (0.8%), or belonging to other religions. Only 28.9% reported actively practicing their religion.

In terms of academic discipline, most respondents were enrolled in health-related fields (70.2%), followed by humanities (13.1%), engineering (8.3%), and science (5.8%). Students were distributed across academic years, with the largest proportions in the first (23.2%) and second years (41.9%).

**Table 7.** Socio-demographic characteristics of the sample (N = 2103 university students).

	N	%
Sex
Male	500	23.80%
Female	1603	76.20%
Age	M = 23.4	SD = 5.68
Young (18–24 years)	1608	76.46%
Young adults (25–39 years)	431	20.45%
Middle age (40–62 years)	65	3.09%
Religious faith
Roman catholic	1103	52.40%
Other religion	137	6.50%
Atheist/Agnostic	571	27.20%
Indifferent	292	13.90%
Religious practice
No	1496	71.10%
Yes	607	28.90%
Study level
Bachelor's degree	1653	78.60%
Master's degree	450	21.40%
Study course
Health sciences (Nursing, Medicine, Dentistry, Nutrition, Psychology)	1477	70.20%
Science (Mathematics, Physics, Chemistry, Biology, Biotechnology, etc.)	122	5.80%
Humanities (Philosophy, Literature, Communication, Law, Educational sciences, Economics, etc.)	275	13.10%
Engineering	175	8.30%
Other	54	2.60%
Course year
1	488	23.20%
2	881	41.90%
3	396	18.80%
4	120	5.70%
5	147	7.00%
6	71	3.40%
Geographical area of study
North	330	15.70%
Center	1157	55.00%
South	156	7.40%
Major islands	460	21.90%
Private University
No	1717	81.60%
Yes	386	18.40%
Offsite
No	1145	54.45%
Yes	638	30.34%
No answerª	320	15.22%
Scholarship/place in the College of Merit
No	1431	68.05%
Yes	344	16.36%
No answerª	328	15.60%

Note: M = mean; SD = standard deviation. ª = For the subsequent analyses of these two variables, only the “No” and “Yes” responses were considered.

Geographically, most participants came from Central Italy (55%), with further representation from the major islands (21.9%), Northern Italy (15.7%), and Southern Italy (7.4%). The majority attended public universities (81.6%), whereas 18.4% studied at private institutions. Additionally, 30.34% were classified as offsite students, defined as students who relocate from their home city to pursue university studies elsewhere, typically incurring higher accommodation and travel expenses. A further 16.36% reported receiving a scholarship or holding a place in a College of Merit. Colleges of Merit are residential institutions recognized by the Ministry of University and Research that admit high-performing students through a competitive selection process based on academic excellence and personal motivation, both of which must be consistently maintained to retain their place in the program. Both offsite status and scholarship/College of Merit placement may increase the pressure on students to sustain high academic performance.

### 3.2. Roots responses

**Figure 2.**
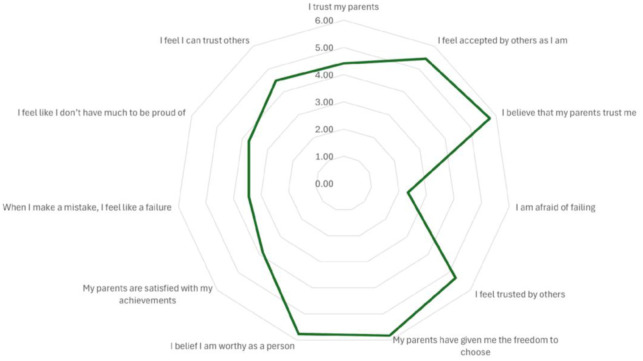
Graphical representation of Roots scores for the whole sample (N = 2103 university students).

The descriptive statistics of the Roots questionnaire (see Table 2 and Figure 2) revealed that students generally reported positive family relationships. The highest mean scores were observed for the items “I perceived myself as receiving love from my parents” (M = 5.94, SD = 1.42), “I believe that my parents trust me” (M = 5.76, SD = 1.42), and “My parents have given me the freedom to choose” (M = 5.83, SD = 1.40), highlighting perceived love, trust, and autonomy within the family context.

Regarding self-perception, participants reported high scores for “I believe I am worthy as a person” (M = 5.77, SD = 1.36) and moderate scores for “No matter what I do, I feel inadequate” (reversed) (M = 4.05, SD = 1.80), both suggesting healthy self-worth, which may represent protective features against perfectionistic tendencies. However, lower scores for the reversed items “I am afraid of failing” (M = 2.33, SD = 1.60) and “When I make a mistake, I feel like a failure” (M = 3.44, SD = 1.89) indicate that some participants also experience self-critical thoughts and elevated fear of failure.

In terms of social relationships, moderately high scores were found for “I feel accepted by others as I am” (M = 5.45, SD = 2.04) and “I feel I can trust others” (M = 4.50, SD = 1.87), reflecting a positive perception of social trust and acceptance.

At the subscale level, the highest mean was observed for Social Relationships (M = 5.10, SD = 1.12), closely followed by Relationships with Family (M = 5.06, SD = 1.01). Relationships with the Self scored the lowest (M = 3.75, SD = 1.15). The weighted mean of the overall Root scale, reflecting the overall relational and emotional background associated with the development of perfectionism, was 4.58 (SD = 0.89; possible range 1–7), and the average total Roots score was 73.20 (SD = 14.30; possible range 16–112). These findings suggest that while students perceive strong parental support, trust, and autonomy, more vulnerable aspects emerge in self-related dimensions, particularly feelings of failure, which may be relevant in understanding the relational and psychological roots of perfectionistic traits.

**Figure 3.**
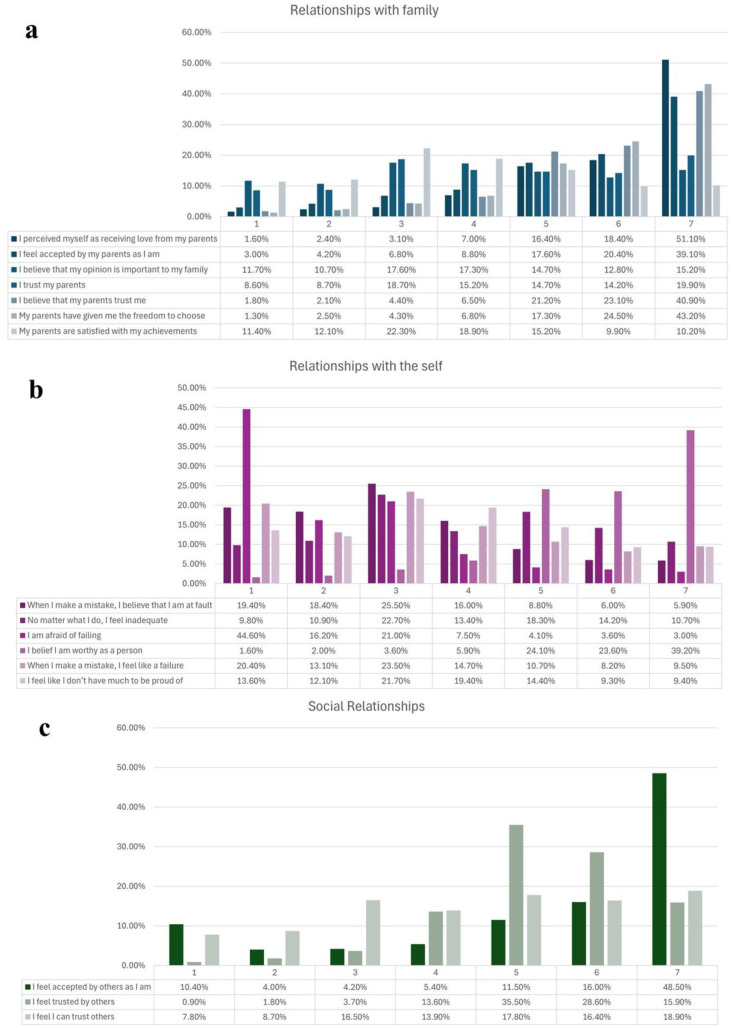
Distribution of responses for the dimensions of Roots: Relationships with Family (a), Relationships with the Self (b), and Social Relationships (c).

Figure 3a–c presents the distribution of responses for the three dimensions of the roots of perfectionism: Relationships with Family (Figure 3a), Relationships with the Self (Figure 3b), and Social Relationships (Figure 3c).

For Relationships with Family, the bar chart shows that most students reported high levels of perceived parental love, with 51.1% selecting the maximum score (7) on the Likert scale. Similarly, a large proportion indicated strong agreement with the statements “I feel accepted by my parents as I am” (39.1%), “My parents have given me the freedom to choose” (43.2%), and “I believe that my parents trust me” (40.9%). By contrast, fewer respondents fully agreed with “I believe that my opinion is important to my family” (15.2%) and “My parents are satisfied with my achievements” (10.2%). Overall, the distribution was skewed toward the higher end of the scale, indicating generally positive perceptions of family relationships.

In terms of Relationships with the Self (Figure 3b), students expressed strong agreement with “I believe I am worthy as a person” (39.2% scored 7). However, they also reported relatively high agreement with the reversed negative items “I am afraid of failing” (most responses clustered at the lower end of the scale), “No matter what I do, I feel inadequate”, and “When I make a mistake, I feel like a failure”. These results suggest that many participants perceive themselves as having a healthy sense of worth, though some still endorse feelings of inadequacy or self-criticism.

Finally, the chart on Social Relationships (Figure 3c) revealed a different response pattern. Nearly half of the students strongly agreed with “I feel accepted by others as I am” (48.5% at score 7). For “I feel trusted by others”, the highest percentages were observed at scores 5 (35.5%) and 6 (28.6%), indicating moderate-to-high trust perceptions. In contrast, “I feel I can trust others” was more evenly distributed, with the highest endorsements at score 5 (17.8%) and score 7 (18.9%), but without peaks observed for other items. These results suggest that students generally felt accepted and trusted by others, but their own trust in others appeared somewhat less consistent.

### 3.3. Comparison between Roots scores and socio-demographic variables

Tables 8 and 9 present the variation of Roots scores across socio-demographic variables such as age and location of study. Younger participants generally reported higher scores in perceived parental love, acceptance, and trust compared to middle-aged respondents. For instance, the rating for “I perceived myself as receiving love from my parents” declined from younger students (M = 6.01) to middle-aged students (M = 5.45; p < 0.001). Conversely, middle-aged individuals reported significantly lower levels of self-critical tendencies, as reflected in lower mean scores for the reversed items “I am afraid of failing” (M = 4.31 vs. 2.19 in younger students) and “When I make a mistake, I feel like a failure” (M = 4.84 vs. 3.35; both p < 0.001).

Geographic differences were also evident. At the composite level, the highest total Roots scores were found among middle-aged participants (M = 80.9, SD = 15.0) and among students from the South and Islands (both M = 76.7, p < 0.001), pointing to notable age- and region-related patterns in relational and self-perception dimensions. Students from the South and the Islands consistently scored higher on family-related support, such as parental love, trust, and freedom of choice (all p < 0.05). For example, mean scores for “I perceived myself as receiving love from my parents” were 6.06 in the South and 6.12 in the Islands, compared to 5.77 in the North and 5.89 in the Center (p < 0.001). On the contrary, students from the Center tended to report lower scores in several family-related domains.

Overall, the composite scores reflected these trends. The score for the Relationships with Family scale was highest in the South (40.60; mean = 5.80) and Islands (40.40; mean = 5.77) and lowest in the middle-aged group (37.8; mean = 5.40). Relationships with the Self showed a significant drop in middle-aged students (22.1; mean = 3.68), highlighting possible developmental or contextual stressors. Finally, the overall Roots score was highest among younger students and in southern regions, underscoring the role of age and family culture in shaping perfectionism. However, it should be noted that the magnitude of these effects is small (effect sizes <0.10), so the observed differences, while consistent, are of limited practical size.

Tables 10 and 11 compare Roots scores across sex, religion, and religious practice. Students identifying as Catholic reported significantly higher scores on most family-related items, including perceived parental love (M = 6.21) and parental trust (M = 5.99), compared to atheist/agnostic (M = 5.59 and 5.44, respectively) and indifferent students (M = 5.76 and 5.67; p < 0.001 in most cases). Catholics also showed the highest composite scores in Relationships with Family (M = 36.7; mean = 5.24) and in the overall Roots scale (M = 75.3; mean = 4.71; p < 0.001). Similarly, students who practiced their religion had significantly higher means in perceived parental love (M = 6.13 vs. 5.86), parental trust (M = 5.94 vs. 5.69), and overall family relationships (M = 36.4; mean = 5.20 vs. 35.0; mean = 5.00; all p < 0.01) compared to non-practicing peers. However, despite these statistically significant differences, the effect sizes were small, indicating that the practical impact of religion and religious practice on these scores is modest.

Regarding sex differences, men reported higher scores in several family support indicators, such as parental trust (M = 4.82 vs. 4.28) and satisfaction of parents with their achievements (M = 4.14 vs. 3.75; both p < 0.001). Moreover, women scored lower on self-related protective constructs such as those regarding fear of failure (M = 2.20 vs. 2.76), identifying mistakes with being a failure (M = 3.30 vs. 3.91; all p < 0.001) and tendency to feel inadequate (M = 3.95 vs. 4.37). These items are reverse-coded, so lower values indicate lower self-compassion. At the composite level, women showed higher scores than men in Relationships with the Self (M = 23.4; mean = 3.90 vs. 21.9; mean = 3.65; p < 0.001), while men scored higher in overall Roots (M = 76.2; mean = 4.76 vs. 72.2; mean = 12.03; p < 0.001). Again, although these differences are statistically significant, the effect sizes are small, suggesting that the practical differences between sexes are limited.

Tables 11 and 12 present comparisons of Roots scores by area of study and level of study. Significant differences were found across academic fields, particularly in items related to parental trust, self-worth, and perfectionism. Students in Health Sciences reported higher protective scores on family-related items, such as “I perceive myself as receiving love from my parents” (M = 5.98) and “I trust my parents” (M = 4.48), compared to those in Science and Engineering. Conversely, Science and Humanities students reported higher perceived acceptance by others (M = 6.09 and 6.07, respectively), while Engineering students showed the lowest means in several indicators, including “I believe that my parents trust me” (M = 5.65) and Relationships with the Self (M = 20.50; mean = 3.42). Students in the “Other” category displayed the highest overall Roots scores (M = 76.10; mean = 4.76), along with greater self-worth (M = 5.96). However, although these differences are statistically significant, the effect sizes are low, indicating that the practical impact of the area of study on these scores is limited.

Regarding level of study, Bachelor's and Master's students scored similarly in most items, with no significant difference in total Roots scores. However, Master's students reported higher trust by others (M = 5.39 vs. 5.28, p = 0.016), greater parental satisfaction with achievements (M = 3.98 vs. 3.81, p = 0.045), and more positive self-perceptions, in the reverse-coded item “I feel like I don't have much to be proud of” (M = 4.05 vs. 3.66, p < 0.001). However, although these differences are statistically significant, the effect sizes are low, indicating that the practical impact of the level of study on these scores is limited.

Overall, these findings suggest that the area of study plays a more decisive role than the level of study in shaping family and self-related protective factors within the Roots framework, although the actual impact of these differences is limited.

Table 13 compares Roots scores across type of university, housing situation, and scholarship or College of Merit placement. Students from private universities consistently reported higher scores in family-related dimensions, such as perceived parental love (M = 6.27 vs. 5.86, p < 0.001), acceptance (M = 5.86 vs. 5.44, p < 0.001), parental trust (M = 6.06 vs. 5.69, p < 0.001), and satisfaction of parents with their achievements (M = 4.36 vs. 3.73, p < 0.001) and the overall Relationships with Family (M = 37.0; mean = 5.29 vs. 35.1; mean = 5.01, p < 0.001). No differences were observed in overall Roots scores. Although these associations reached statistical significance, effect sizes were generally small across all variables—with the exception of “I feel accepted by others as I am”, which showed a moderate effect size, indicating a more substantive practical difference.

**Table 8.** Comparison between Roots scores, age, and location of study.

	Whole sample		Age								Location
	
			Young (Y)		Young adults (YA)		Middle age (MA)				North (N)		Center (C)		South (SO)		Islands (I)	
								
	M	SD	M	SD	Mn	SD	M	SD	p-value^&^	ε²	M	SD	M	SD	M	SD	M	SD	p-value^&^	ε²
I perceived myself as receiving love from my parents	5.94	1.42	6.01	1.38	5.74	1.53	5.45	1.64	<0.001	0.009	5.77	1.53	5.89	1.41	6.06	1.44	6.12	1.34	<0.001	0.009
When I make a mistake, I believe that I am at fault	3.18	1.71	3.13	1.67	3.23	1.80	4.03	1.93	0.001	0.006	3.08	1.59	3.07	1.71	3.26	1.78	3.51	1.73	<0.001	0.013
I feel accepted by my parents as I am	5.52	1.65	5.57	1.64	5.35	1.69	5.14	1.75	0.003	0.006	5.49	1.63	5.42	1.70	5.82	1.50	5.68	1.57	0.002	0.007
I believe that my opinion is important to my family	4.12	1.92	4.08	1.88	4.15	2.01	4.89	2.02	0.003	0.006	4.12	1.81	3.99	1.99	4.56	1.80	4.28	1.80	0.001	0.008
No matter what I do, I feel inadequate	4.05	1.80	3.99	1.78	4.19	1.86	4.59	1.87	0.005	0.005	4.03	1.75	3.96	1.83	4.26	1.90	4.21	1.72	0.040	0.004
I trust my parents	4.41	1.91	4.33	1.90	4.55	1.94	5.48	1.73	<0.001	0.013	4.27	1.82	4.28	1.95	4.61	1.88	4.75	1.85	<0.001	0.011
I feel accepted by others as I am	5.45	2.04	5.38	2.09	5.69	1.85	5.67	1.88	0.068	0.003	6.04	1.39	4.94	2.35	6.09	1.33	6.12	1.25	<0.001	0.048
I believe that my parents trust me	5.76	1.42	5.81	1.39	5.64	1.46	5.48	1.69	0.037	0.003	5.67	1.39	5.70	1.46	6.01	1.28	5.90	1.35	0.002	0.007
I am afraid of failing	2.33	1.60	2.19	1.48	2.58	1.73	4.31	2.01	<0.001	0.041	2.29	1.51	2.21	1.56	2.28	1.60	2.68	1.70	<0.001	0.016
I feel trusted by others	5.31	1.19	5.29	1.19	5.33	1.20	5.58	1.12	0.122	0.002	5.12	1.11	5.32	1.24	5.38	1.19	5.38	1.11	0.003	0.007
My parents have given me the freedom to choose	5.83	1.40	5.86	1.36	5.75	1.46	5.41	1.86	0.178	0.002	5.68	1.41	5.81	1.44	6.01	1.26	5.92	1.34	0.018	0.005
I believe I am worthy as a person	5.77	1.36	5.74	1.36	5.83	1.37	6.05	1.30	0.043	0.003	5.66	1.32	5.69	1.47	6.04	1.07	5.95	1.15	0.002	0.007
My parents are satisfied with my achievements	3.85	1.78	3.81	1.76	3.93	1.85	4.33	1.79	0.050	0.003	3.51	1.75	3.85	1.80	4.06	1.82	4.00	1.72	<0.001	0.009
When I make a mistake, I feel like a failure	3.44	1.89	3.35	1.82	3.60	2.02	4.84	2.07	<0.001	0.016	3.30	1.77	3.34	1.96	3.74	1.89	3.71	1.77	<0.001	0.011
I feel like I don't have much to be proud of	3.74	1.80	3.66	1.77	3.95	1.85	4.39	1.87	<0.001	0.007	3.58	1.69	3.83	1.88	3.79	1.73	3.63	1.64	0.094	0.003
I feel I can trust others	4.50	1.87	4.46	1.83	4.55	1.99	5.28	1.94	<0.001	0.007	4.40	1.78	4.37	1.92	4.71	1.85	4.83	1.78	<0.001	0.011
Relationships with Family	35.40	7.06	35.50	6.95	35.10	7.39	36.20	7.30	0.484	0.001	34.50	6.98	34.90	7.08	37.10	6.60	36.60	6.96	<0.001	0.016
Relationships with the Self	22.50	6.89	22.10	6.60	23.40	7.48	28.20	6.93	<0.001	0.023	21.90	6.84	22.10	6.85	23.40	7.28	23.70	6.75	<0.001	0.011
Social Relationships	15.30	3.37	15.10	3.34	15.60	3.40	16.50	3.59	<0.001	0.008	15.60	2.95	14.60	3.60	16.20	2.92	16.30	2.79	<0.001	0.045
Roots	73.20	14.30	72.60	13.90	74.10	15.30	80.90	15.00	<0.001	0.010	72.00	14.20	71.70	14.20	76.70	14.50	76.70	13.90	<0.001	0.026

Note: M = Mean. SD = Standard deviation. & = Kruskal–Wallis test.

**Table 9.** Comparison among p-values of Roots scores for age and location.

	Age			Location					
	
	Y vs. YA	Y vs. MA	YA vs MA	N vs. C	N vs. SO	N vs. I	C vs. SO	C vs. I	SO vs. I
I perceived myself as receiving love from my parents	0.001	0.011	0.359	0.682	0.065	0.002	0.173	0.003	0.999
When I make a mistake, I believe that I am at fault	0.834	<0.001	0.005	0.933	0.774	0.001	0.514	<0.001	0.332
I feel accepted by my parents as I am	0.010	0.085	0.648	0.963	0.087	0.277	0.017	0.025	0.702
I believe that my opinion is important to my family	0.766	0.002	0.014	0.737	0.064	0.509	0.005	0.037	0.383
No matter what I do, I feel inadequate	0.103	0.017	0.205	0.922	0.544	0.525	0.214	0.071	0.975
I trust my parents	0.060	<0.001	<0.001	0.999	0.247	0.001	0.211	<0.001	0.820
I feel accepted by others as I am	0.071	0.625	1.000	<0.001	0.996	0.975	<0.001	<0.001	1.000
I believe that my parents trust me	0.052	0.404	0.936	0.846	0.026	0.035	0.052	0.041	0.877
I am afraid of failing	<0.001	<0.001	<0.001	0.459	0.968	0.005	0.961	<0.001	0.022
I feel trusted by others	0.637	0.137	0.299	0.003	0.045	0.008	0.981	0.997	0.994
My parents have given me the freedom to choose	0.453	0.281	0.593	0.151	0.041	0.029	0.499	0.636	0.936
I believe I am worthy as a person	0.267	0.091	0.338	0.553	0.013	0.009	0.100	0.071	0.895
My parents are satisfied with my achievements	0.592	0.050	0.191	0.010	0.010	<0.001	0.615	0.461	0.994
When I make a mistake, I feel like a failure	0.130	<0.001	<0.001	0.997	0.073	0.007	0.038	<0.001	0.999
I feel like I don't have much to be proud of	0.013	0.008	0.214	0.170	0.688	0.981	0.993	0.222	0.819
I feel I can trust others	0.412	<0.001	0.012	0.999	0.237	0.003	0.166	<0.001	0.926
Relationships with family	0.723	0.656	0.529	0.501	<0.001	<0.001	0.005	<0.001	0.966
Relationships with the self	0.005	<0.001	<0.001	0.974	0.187	0.001	0.182	<0.001	0.940
Social relationships	0.035	0.002	0.052	<0.001	0.120	0.002	<0.001	<0.001	0.971
Roots	0.239	<0.001	0.003	0.995	0.007	<0.001	<0.001	<0.001	1.000

Note: Y = Young. YA = Young adults. MA = Middle-aged. N = North. C = Center. SO = South. I = Islands. Comparison using the Dwass–Steel–Critchlow–Fligner pairwise test.

**Table 10.** Comparison between Roots scores, sex, religion, and religious practice.

	Religion										Practicing their religion						Sex					
	
	Catholic (CA)		Other religion (O)		Atheist/Agnostic (A)		Indifferent (I)				No		Yes				Man		Woman	
									
	M	SD	M	SD	M	SD	M	SD	p-value^&^	ε²	M	SD	M	SD	p-value^$^	r	M	SD	M	SD	p-value^$^	r
I perceived myself as receiving love from my parents	6.21	1.19	5.50	1.76	5.59	1.59	5.76	1.48	<0.001	0.040	5.86	1.48	6.13	1.24	<0.001	0.006	6.05	1.33	5.90	1.45	0.072	0.002
When I make a mistake, I believe that I am at fault	3.27	1.72	3.07	1.65	2.99	1.61	3.25	1.83	0.012	0.005	3.11	1.70	3.34	1.71	0.002	0.004	3.57	1.78	3.06	1.67	<0.001	0.016
I feel accepted by my parents as I am	5.83	1.50	5.29	1.75	5.04	1.74	5.35	1.72	<0.001	0.049	5.41	1.71	5.77	1.47	<0.001	0.008	5.63	1.56	5.48	1.68	0.134	0.001
I believe that my opinion is important to my family	4.23	1.92	4.09	1.89	3.92	1.89	4.06	1.93	0.010	0.005	4.09	1.92	4.17	1.92	0.336	0.000	4.20	1.93	4.09	1.91	0.291	0.001
No matter what I do, I feel inadequate	4.12	1.80	4.24	1.84	3.83	1.80	4.13	1.79	0.006	0.006	4.00	1.79	4.18	1.83	0.041	0.002	4.37	1.81	3.95	1.79	<0.001	0.010
I trust my parents	4.59	1.89	4.35	1.90	4.15	1.91	4.25	1.92	<0.001	0.011	4.31	1.91	4.66	1.89	<0.001	0.007	4.82	1.90	4.28	1.90	<0.001	0.015
I feel accepted by others as I am	5.40	2.13	5.53	1.94	5.62	1.84	5.29	2.06	0.277	0.002	5.52	1.99	5.30	2.14	0.093	0.001	5.13	2.07	5.55	2.02	<0.001	0.013
I believe that my parents trust me	5.99	1.28	5.45	1.71	5.44	1.48	5.67	1.47	<0.001	0.034	5.69	1.47	5.94	1.27	0.002	0.005	5.88	1.38	5.72	1.43	0.016	0.003
I am afraid of failing	2.44	1.65	2.61	1.77	2.10	1.44	2.26	1.56	<0.001	0.009	2.24	1.53	2.56	1.72	<0.001	0.007	2.76	1.81	2.20	1.50	<0.001	0.019
I feel trusted by others	5.38	1.15	5.17	1.21	5.24	1.22	5.24	1.27	0.057	0.004	5.27	1.21	5.41	1.14	0.012	0.003	5.32	1.26	5.30	1.17	0.316	0.000
My parents have given me the freedom to choose	5.92	1.33	5.53	1.54	5.77	1.46	5.74	1.47	0.010	0.005	5.81	1.41	5.86	1.38	0.531	0.000	5.87	1.40	5.81	1.40	0.378	0.000
I believe I am worthy as a person	5.89	1.26	5.73	1.54	5.62	1.37	5.60	1.57	<0.001	0.008	5.75	1.37	5.81	1.34	0.314	0.000	5.88	1.43	5.73	1.34	<0.001	0.005
My parents are satisfied with my achievements	3.90	1.77	3.78	1.91	3.70	1.76	3.98	1.80	0.070	0.003	3.82	1.79	3.91	1.76	0.295	0.001	4.14	1.64	3.75	1.81	<0.001	0.009
When I make a mistake, I feel like a failure	3.58	1.90	3.45	1.98	3.18	1.82	3.46	1.91	<0.001	0.008	3.36	1.87	3.66	1.93	<0.001	0.005	3.91	1.95	3.30	1.85	<0.001	0.018
I feel like I don't have much to be proud of	3.84	1.78	3.93	1.81	3.51	1.82	3.74	1.77	0.002	0.007	3.70	1.82	3.86	1.73	0.048	0.002	4.07	1.75	3.64	1.80	<0.001	0.011
I feel I can trust others	4.68	1.83	4.58	1.97	4.16	1.87	4.47	1.90	<0.001	0.014	4.43	1.88	4.67	1.83	0.010	0.003	4.60	1.91	4.47	1.86	0.113	0.001
Relationships with Family	36.70	6.63	34.00	7.90	33.60	7.08	34.80	7.22	<0.001	0.036	35.00	7.23	36.40	6.51	<0.001	0.007	36.60	6.83	35.00	7.09	<0.001	0.008
Relationships with the Self	23.10	6.80	23.00	7.31	21.20	6.68	22.40	7.13	<0.001	0.015	22.20	6.94	23.40	6.69	<0.001	0.007	24.60	6.93	21.90	6.76	<0.001	0.027
Social Relationships	15.50	3.42	15.30	3.49	15.00	3.19	15.00	3.43	0.013	0.005	15.20	3.35	15.40	3.42	0.284	0.001	15.10	3.58	15.30	3.30	0.351	0.000
Roots	75.30	13.90	72.30	15.50	69.90	13.70	72.20	15.10	<0.001	0.026	72.40	14.60	75.20	13.60	<0.001	0.007	76.20	14.50	72.20	14.20	<0.001	0.014

Note: M = Mean. SD = Standard deviation. $ =Mann–Whitney U test. & = Kruskal–Wallis test.

**Table 11.** Comparison among p-values of Roots scores for religion and area of study.

	Religion						Area of study									
	
	CA vs. O	CA vs. A	CA vs. I	O vs. A	O vs. I	A vs. I	HS vs. S	HS vs. H	HS vs. E	HS vs. OT	S vs. H	S vs. E	S vs. OT	H vs. E	H vs. OT	E vs. H
I perceived myself as receiving love from my parents	<0.001	<0.001	<0.001	<0.001	0.662	0.395	0.503	0.030	1.000	0.391	0.998	0.647	0.989	0.224	0.996	0.457
When I make a mistake, I believe that I am at fault	0.512	0.007	0.959	0.968	0.854	0.300	0.546	1.000	<0.001	0.961	0.564	0.547	0.708	0.002	0.990	0.060
I feel accepted by my parents as I am	0.001	<0.001	<0.001	0.341	0.991	0.027	0.351	0.018	0.891	0.997	0.998	0.918	0.955	0.671	0.867	1.000
I believe that my opinion is important to my family	0.853	0.005	0.501	0.685	0.996	0.723	1.000	0.999	0.758	0.960	1.000	0.921	0.985	0.782	0.990	0.787
No matter what I do, I feel inadequate	0.862	0.010	0.998	0.076	0.935	0.085	0.995	0.689	0.327	0.918	0.801	0.522	0.988	0.961	0.648	0.441
I trust my parents	0.503	<0.001	0.032	0.645	0.953	0.866	0.111	0.856	0.031	0.793	0.590	1.000	0.167	0.436	0.592	0.107
I feel accepted by others as I am	0.991	0.904	0.447	0.999	0.598	0.190	0.068	<0.001	0.002	0.778	0.979	<0.001	0.982	<0.001	0.837	0.025
I believe that my parents trust me	0.003	<0.001	0.004	0.799	0.818	0.052	0.071	0.006	0.641	0.986	1.000	0.780	0.359	0.762	0.342	0.774
I am afraid of failing	0.758	<0.001	0.306	0.010	0.212	0.619	0.465	0.519	0.008	0.340	0.978	0.939	0.111	0.460	0.126	0.008
I feel trusted by others	0.219	0.145	0.432	0.911	0.912	1.000	0.999	0.435	0.951	0.817	0.770	0.999	0.860	0.430	0.999	0.694
My parents have given me the freedom to choose	0.013	0.291	0.351	0.217	0.409	0.995	1.000	0.644	0.485	0.974	0.939	0.835	0.985	0.996	0.792	0.687
I believe I am worthy as a person	0.944	<0.001	0.101	0.410	0.798	0.920	0.803	0.950	0.012	0.915	0.659	0.771	0.681	0.016	0.992	0.123
My parents are satisfied with my achievements	0.820	0.111	0.883	0.987	0.677	0.127	0.856	0.899	0.775	0.696	0.999	1.000	0.420	0.998	0.509	0.410
When I make a mistake, I feel like a failure	0.846	<0.001	0.726	0.515	0.999	0.187	0.040	1.000	<0.001	0.994	0.125	0.984	0.357	0.007	0.998	0.122
I feel like I don't have much to be proud of	0.949	0.002	0.871	0.068	0.777	0.235	0.993	0.362	0.750	0.718	0.632	0.824	0.943	1.000	0.317	0.525
I feel I can trust others	0.982	<0.001	0.394	0.078	0.911	0.080	0.264	0.999	<0.001	1.000	0.367	0.913	0.885	0.010	0.999	0.421
Relationships with family	0.001	<0.001	<0.001	0.833	0.874	0.092	0.342	0.122	0.147	1.000	0.999	1.000	0.695	1.000	0.780	0.725
Relationships with the self	0.987	<0.001	0.430	0.041	0.901	0.068	0.484	0.801	<0.001	0.742	0.962	0.738	0.379	0.119	0.552	0.015
Social relationships	0.978	0.019	0.133	0.605	0.723	1.000	0.977	0.001	<0.001	0.727	0.441	0.002	0.960	<0.001	0.986	0.004
Roots	0.299	<0.001	0.018	0.124	0.994	0.083	0.466	0.956	<0.001	0.865	0.906	0.768	0.413	0.039	0.779	0.025

Note: CA = Catholic. O = Other religions. A = Atheist/Agnostic. I = Indifferent. HS = Health Science. S = Science. H = Humanities. E = Engineering. OT = Others. Comparison using the Dwass–Steel–Critchlow–Fligner pairwise test.

**Table 12.** Comparison between Roots scores, area, and level of study.

	Area of study												Level of study					
	
	Health science (HS)		Science (S)		Humanities (H)		Engineering (E)		Others (OT)				Bachelor's degree		Master's degree	
						
	M	SD	M	SD	M	SD	M	SD	M	SD	p-value^&^	ε²	M	SD	M	SD	p-value^$^	r
I perceived myself as receiving love from my parents	5.98	1.40	5.73	1.60	5.76	1.45	6.01	1.39	5.70	1.45	0.012	0.006	5.92	1.42	5.97	1.42	0.423	0.000
When I make a mistake, I believe that I am at fault	3.23	1.70	3.05	1.83	3.28	1.74	2.67	1.55	3.39	1.81	<0.001	0.010	3.17	1.72	3.24	1.68	0.419	0.000
I feel accepted by my parents as I am	5.60	1.60	5.32	1.70	5.22	1.81	5.46	1.72	5.44	1.79	0.018	0.006	5.52	1.64	5.49	1.69	0.832	0.000
I believe that my opinion is important to my family	4.12	1.88	4.13	2.06	4.16	2.00	3.94	1.98	4.28	1.91	0.721	0.001	4.12	1.89	4.11	1.99	0.996	0.000
No matter what I do, I feel inadequate	4.08	1.80	4.16	1.84	3.93	1.80	3.82	1.79	4.28	1.88	0.190	0.003	4.04	1.79	4.06	1.86	0.916	0.000
I trust my parents	4.48	1.89	4.05	2.01	4.35	1.94	4.03	1.93	4.78	1.88	0.004	0.007	4.38	1.91	4.53	1.90	0.140	0.001
I feel accepted by others as I am	5.36	2.11	6.09	1.23	6.07	1.44	4.70	2.40	5.93	1.48	<0.001	0.022	5.72	1.81	4.48	2.47	<0.001	0.043
I believe that my parents trust me	5.84	1.35	5.48	1.58	5.51	1.54	5.65	1.55	5.83	1.55	0.001	0.009	5.74	1.43	5.84	1.37	0.210	0.001
I am afraid of failing	2.37	1.58	2.22	1.66	2.27	1.64	2.04	1.54	2.76	1.70	<0.001	0.009	2.32	1.60	2.38	1.57	0.260	0.001
I feel trusted by others	5.31	1.14	5.20	1.35	5.38	1.29	5.19	1.27	5.41	1.35	0.299	0.002	5.28	1.18	5.39	1.22	0.016	0.003
My parents have given me the freedom to choose	5.88	1.32	5.79	1.55	5.65	1.61	5.63	1.59	5.93	1.40	0.329	0.002	5.82	1.40	5.84	1.40	0.830	0.000
I believe I am worthy as a person	5.81	1.31	5.57	1.61	5.83	1.38	5.41	1.54	5.96	1.21	0.009*	0.006	5.76	1.35	5.78	1.40	0.539	0.000
My parents are satisfied with my achievements	3.87	1.78	3.75	1.83	3.77	1.80	3.72	1.81	4.17	1.62	0.319	0.002	3.81	1.80	3.98	1.72	0.045	0.002
When I make a mistake, I feel like a failure	3.51	1.86	3.09	1.98	3.55	1.96	2.94	1.85	3.65	2.00	<0.001	0.011	3.44	1.88	3.47	1.93	0.825	0.000
I feel like I don't have much to be proud of	3.77	1.78	3.84	1.85	3.57	1.82	3.61	1.84	4.06	1.83	0.159	0.003	3.66	1.77	4.05	1.87	<0.001	0.008
I feel I can trust others	4.57	1.83	4.19	2.04	4.59	1.90	3.99	1.89	4.50	2.02	0.001	0.009	4.47	1.87	4.61	1.87	0.155	0.001
Relationships with Family	35.80	6.76	34.20	8.01	34.40	8.01	34.40	6.83	36.10	7.43	0.017	0.006	35.30	7.08	35.80	6.96	0.119	0.001
Relationships with the Self	22.80	6.69	21.90	8.00	22.40	7.60	20.50	6.09	24.10	7.33	<0.001	0.010	22.40	6.98	23.00	6.54	0.073	0.002
Social Relationships	15.20	3.34	15.50	3.35	16.00	3.30	13.90	3.43	15.80	3.28	<0.001	0.022	15.50	3.26	14.50	3.65	<0.001	0.013
Roots	73.80	13.80	71.60	17.20	72.90	16.30	68.80	12.40	76.10	15.20	<0.001	0.011	73.20	14.50	73.20	13.90	0.964	0.000

Note: M = Mean. SD = Standard deviation. $ = Mann–Whitney U test. & = Kruskal–Wallis test.

**Table 13.** Comparison between Roots scores, private university, offsite study, and scholarship/place in College of Merit.

	Private University						Offsite						Scholarship/place in the College of Merit					
	
	No		Yes				No		Yes				No		Yes	
									
	M	SD	M	SD	p-value^$^	r	M	SD	M	SD	p-value^$^	r	M	SD	M	SD	p-value^$^	r
I perceived myself as receiving love from my parents	5.86	1.47	6.27	1.10	<0.001	0.011	5.80	1.51	6.02	1.38	0.002	0.011	5.88	1.46	5.87	1.49	0.939	0.006
When I make a mistake, I believe that I am at fault	3.19	1.70	3.15	1.76	<0.001	0.000	3.15	1.68	3.29	1.75	0.093	0.002	3.13	1.68	3.48	1.78	0.001	0.006
I feel accepted by my parents as I am	5.44	1.69	5.86	1.45	<0.001	0.010	5.39	1.70	5.58	1.64	0.017	0.010	5.42	1.69	5.64	1.64	0.012	0.009
I believe that my opinion is important to my family	4.14	1.90	4.01	2.00	<0.001	0.000	4.17	1.91	4.18	1.86	0.239	0.004	4.16	1.90	4.21	1.85	0.475	0.004
No matter what I do, I feel inadequate	4.04	1.78	4.06	1.91	<0.001	0.000	4.00	1.78	4.17	1.80	0.029	0.002	4.07	1.79	4.02	1.78	0.034	0.000
I trust my parents	4.39	1.89	4.48	1.98	<0.001	0.000	4.35	1.87	4.54	1.94	<0.001	0.002	4.37	1.89	4.61	1.93	0.214	0.002
I feel accepted by others as I am	6.10	1.32	2.58	2.19	0.960	0.280	6.08	1.33	6.10	1.35	0.134	0.345^††^	6.09	1.34	6.06	1.36	0.061	0.336^††^
I believe that my parents trust me	5.69	1.45	6.06	1.23	<0.001	0.011	5.65	1.46	5.82	1.40	0.009	0.011	5.68	1.44	5.81	1.44	0.045	0.009
I am afraid of failing	2.35	1.61	2.27	1.54	<0.001	0.000	2.31	1.57	2.41	1.66	0.322	0.001	2.31	1.57	2.49	1.74	0.172	0.001
I feel trusted by others	5.31	1.17	5.28	1.26	0.912	0.000	5.32	1.15	5.33	1.21	0.473	0.001	5.30	1.16	5.39	1.18	0.191	0.001
My parents have given me the freedom to choose	5.81	1.42	5.92	1.32	0.238	0.001	5.79	1.44	5.84	1.39	0.687	0.001	5.78	1.43	5.94	1.36	0.044	0.002
I believe I am worthy as a person	5.76	1.33	5.80	1.48	0.121	0.001	5.79	1.30	5.79	1.35	0.705	0.000	5.77	1.31	5.85	1.34	0.184	0.001
My parents are satisfied with my achievements	3.73	1.76	4.36	1.79	<0.001	0.020	3.67	1.76	3.94	1.77	0.713	0.020	3.72	1.76	3.92	1.77	<0.001	0.017
When I make a mistake, I feel like a failure	3.42	1.87	3.55	2.00	<0.001	0.000	3.35	1.84	3.63	1.91	0.003	0.004	3.40	1.86	3.65	1.90	0.021	0.003
I feel like I don't have much to be proud of	3.59	1.75	4.44	1.84	<0.001	0.033	3.56	1.74	3.69	1.75	0.201	0.033	3.58	1.75	3.65	1.70	<0.001	0.036
I feel I can trust others	4.48	1.87	4.59	1.88	0.908	0.000	4.46	1.86	4.57	1.89	0.105	0.001	4.43	1.87	4.82	1.84	0.154	0.006
Relationships with Family	35.10	7.33	37.00	5.39	<0.001	0.009	34.80	7.25	35.90	7.46	0.013	0.009	35.00	7.37	36.00	7.16	0.048	0.006
Relationships with the Self	22.30	7.20	23.30	5.25	0.804	0.005	22.20	7.07	23.00	7.41	0.011	0.005	22.30	7.22	23.10	7.19	<0.001	0.004
Social Relationships	15.90	3.01	12.40	3.47	0.915	0.139	15.90	2.99	16.00	3.06	0.902	0.190	15.80	3.02	16.30	2.98	0.422	0.185
Roots	73.30	15.00	72.70	10.90	<0.001	0.001	72.80	14.70	74.90	15.50	0.932	0.009	73.10	15.10	75.40	14.60	0.574	0.008

Note: M = Mean. SD = Standard deviation. $ = Mann–Whitney U test. ^††^ = Significant differences indicated by effect size r > 0.3.

Students living offsite showed higher means than students living in their cities in some family- and self-related domains, namely “I believe that my parents trust me” (M = 5.82 vs. 5.65, p = 0.009), “No matter what I do, I feel inadequate” (reverse-coded) (M = 4.17 vs. 4.00, p = 0.029), and Relationships with the Self (M = 23.0; mean = 3.83 vs. 22.2; mean = 3.70, p = 0.001). Students with scholarships or College of Merit placements also reported higher protective features against perfectionism than their counterparts. They scored significantly higher on reverse-coded items such as “When I make a mistake, I feel like a failure” (M = 3.65 vs. 3.40, p = 0.021) and “I feel like I don't have much to be proud of” (M = 3.65 vs. 3.58, p < 0.001) and slightly higher scores in family relationships (M = 36.0; mean = 6.00 vs. 35.0; mean = 5.83, p = 0.048). However, they also reported lower parental satisfaction with achievements (M = 3.92 vs. 3.72, p < 0.001). As with university type, effect sizes for these comparisons were consistently small, again with the single exception of the item “I feel accepted by others as I am”, which exhibited a moderate effect size.

No significant group differences emerged in Social Relationships, and total Roots scores did not differ between offsite students or scholarship holders and their counterparts.

### 3.4. Clustering analysis of the Roots variables

**Figure 4.**
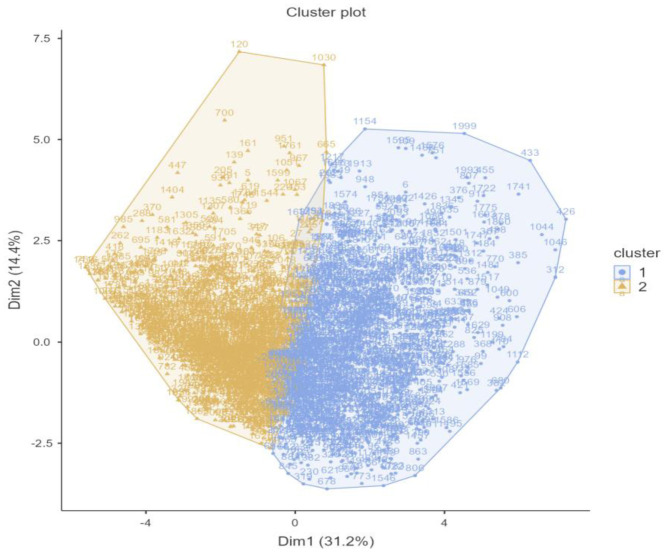
Cluster plot.

The k-means clustering analysis was performed on the 16 continuous variables of the Roots scale (Figure 4). The solution with two clusters was retained based on the elbow plot (Figure 1). To evaluate the quality of the two-cluster solution, a sum of squares analysis was conducted. The total sum of squares (Total SS), representing the overall variance in the dataset, was 95,987. Of this, 20,382 was attributed to between-cluster variance, reflecting the variability explained by the separation between clusters. The remaining variance, 42,433 in Cluster 1 (n = 1096) and 33,173 in Cluster 2 (n = 1007), was accounted for by within-cluster variation.

The proportion of variance explained by the clustering solution (i.e., the ratio of between-cluster to total sum of squares) was approximately 21.2%, suggesting a modest differentiation between the two clusters. Although this percentage indicates that a considerable amount of heterogeneity remains within clusters, such values are not uncommon in behavioral and social science research, where complex constructs and multifactorial influences often limit the degree of variance that clustering can capture. The optimal partition yielded a mean silhouette score of 0.182, reflecting weak separation and limited internal cohesion among clusters. Consequently, the findings should be regarded as exploratory in nature, offering preliminary insights rather than supporting the existence of a stable or robust typology.

**Figure 5.**
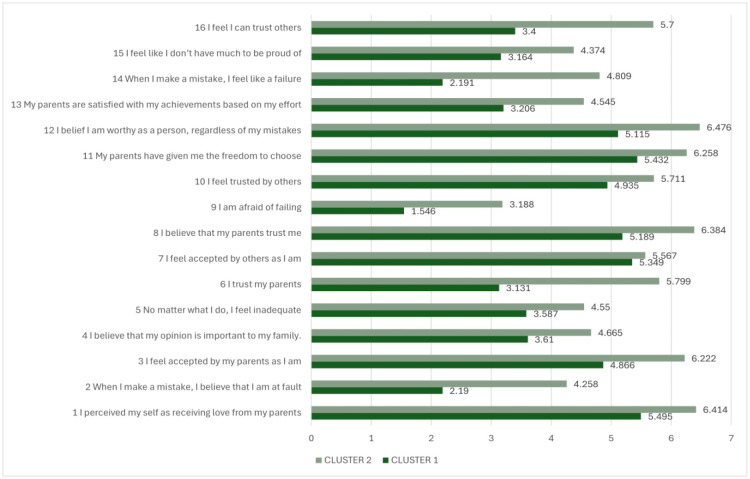
Mean scores of the Roots items for the two identified clusters.

The bar chart (Figure 5) compares the responses to self-perception and family/social-related items between the two clusters identified through k-means analysis. Overall, Cluster 1 consistently reported higher mean scores across nearly all positive items, indicating stronger feelings of parental support, trust, and self-worth, whereas Cluster 2 reported less negative self-perceptions (e.g., inadequacy, fear of failure, self-blame).

Specifically, Cluster 2 participants expressed higher agreement with statements such as “I perceived myself as receiving love from my parents” (M = 6.41 vs. 5.50), “I believe I am worthy as a person, regardless of my mistakes” (M = 6.48 vs. 5.12), and “I believe that my parents trust me” (M = 6.38 vs. 5.19). Similarly, they scored higher on items related to acceptance, such as “I feel accepted by my parents as I am” (M = 6.22 vs. 4.87) and “I feel accepted by others as I am” (M = 5.80 vs. 5.35). Cluster 1 also reported greater trust toward both parents (M = 5.80 vs. 3.13) and others (M = 5.71 vs. 4.94).

Cluster 2 also showed higher self-related protective constructs with stronger endorsement of reverse-coded items such as “When I make a mistake, I feel like a failure” (M = 4.81 vs. 2.19), “No matter what I do, I feel inadequate” (M = 4.55 vs. 3.59), and “I am afraid of failing” (M = 3.19 vs. 1.55), “When I make a mistake, I believe that I am at fault” (M = 4.26 vs. 2.19), and feelings of lacking pride in themselves (M = 4.37 vs. 3.16). Together, these findings suggest that Cluster 2 reflects individuals with stronger parental support, higher self-esteem, and greater perceived acceptance from others.

**Table 14.** Sociodemographic profiles of participants in the clusters.

		CLUSTER 1 (n = 1096)	CLUSTER 2 (n = 1007)
			
		%	%	p-value^†^
Sex	Male	19.27	28.17	<0.001
	Female	80.73	71.83	
Age	Young (18–24 years)	77.94	75.02	0.001
	Young adults (25–39 years)	20.42	20.56	
	Middle age (40–62 years)	1.64	4.41	
Religion	Catholic	45.28	59.44	<0.001
	Other religion	6.94	6.10	
	Atheist/agnostic	33.72	20.75	
	Indifferent	14.07	13.71	
Area of study	Health science (Nursing, Medicine, Dentistry, Nutrition, Psychology)	67.15	73.24	0.003
	Science (Mathematics, Physics, Chemistry, Biology, Biotechnology, etc.)	7.23	4.41	
	Humanities (Philosophy, Literature, Communication, Law, Educational sciences, Economics, etc.)	13.20	12.96	
	Engineering	9.92	6.76	
	Others	2.50	2.63	
Level of study	Bachelor's degree	80.15	77.09	0.100
	Master's degree	19.85	22.91	
Location	North	17.15	14.27	<0.001
	Center	58.09	52.02	
	South	6.74	8.08	
	Islands	18.02	25.63	
Private University	No	84.97	78.40	<0.001
	Yes	15.03	21.60	
Offsite	No	58.57	50.42	<0.001
	Yes	28.52	32.21	

Note: ^†^ = Pearson's Chi-squared test with Yates' continuity correction.

A comparison of the two clusters revealed statistically significant differences across several sociodemographic and academic variables (Table 14). Sex distribution differed significantly between the groups (p < 0.001), with Cluster 1 showing a higher proportion of females (80.73%) compared to Cluster 2 (71.83%), whereas males were more represented in Cluster 2 (28.17% vs. 19.27%). Age composition also differed significantly (p = 0.001). Both clusters were primarily composed of young students aged 18–24 years (Cluster 1 = 77.94%, Cluster 2 = 75.02%), but Cluster 2 had a higher proportion of middle-aged participants (40–62 years; 4.41% vs. 1.64%). Religious affiliation showed a highly significant difference (p < 0.001). Cluster 2 contained more Catholics (59.44% vs. 45.28%), whereas Cluster 1 had larger proportions of atheists/agnostics (33.72% vs. 20.75%). The proportions of individuals reporting “other religion” or “indifferent” were similar across clusters. Geographic distribution showed strong differences (p < 0.001). Cluster 2 had a higher proportion of students from the islands (25.63% vs. 18.02%), while Cluster 1 was more represented in the Center (58.09% vs. 52.02%) and North of Italy (17.15% vs. 14.27%).

Regarding the area of study, significant differences were observed (p = 0.003). Cluster 2 had a higher proportion of students in health sciences (73.24% vs. 67.15%), whereas Cluster 1 included relatively more students in science (7.23% vs. 4.41%) and engineering (9.92% vs. 6.76%). Humanities and other fields were comparably represented across groups. No significant differences emerged for level of study (p = 0.100), with both clusters being predominantly composed of bachelor's degree students (~80%). Institutional characteristics also differed significantly. Cluster 1 had a larger proportion of students from public universities (84.97% vs. 78.40%; p < 0.001), while Cluster 2 included more students attending private universities (21.60% vs. 15.03%). Additionally, Cluster 2 comprised more offsite students (32.21% vs. 28.52%; p < 0.001).

### 3.5. LPA results

#### 3.5.1. Profile sizes and Roots score patterns

On this basis, the 4-profile model was retained for subsequent analyses. The four latent profiles were of adequate and interpretable size (Table 15). Profile 1 included n = 749 participants (35.6%), Profile 2 included n = 474 participants (22.5%), Profile 3 included n = 222 participants (10.6%), and Profile 4 included n = 658 participants (31.3%). Using the sum scores of the Roots total scale and of the three subscales (family relationships, relationship with the self, and social relationships), distinct patterns emerged across profiles. Profile 1 showed the lowest overall Roots scores (M = 61.0, SD = 10.3), with comparatively lower family (M = 28.6, SD = 5.51) and self scores (M = 19.1, SD = 5.76), and moderate social scores (M = 13.4, SD = 3.13). Profile 2 was characterized by moderately elevated total Roots scores (M = 70.1, SD = 7.52), high family scores (M = 36.0, SD = 3.83), but relatively lower self scores (M = 18.0, SD = 3.92), with social scores in the mid-to-high range (M = 16.0, SD = 2.32).

Profile 3 showed high overall Roots scores (M = 75.0, SD = 6.66) and very high family (M = 39.0, SD = 3.28) and self scores (M = 24.1, SD = 4.40), but comparatively lower social scores (M = 11.9, SD = 2.10), consistent with strong family and self-related resources alongside weaker perceived social relationships. Finally, Profile 4 exhibited the most favorable pattern across all domains, with very high total Roots scores (M = 88.6, SD = 8.07), as well as high family (M = 41.6, SD = 3.80), self (M = 29.1, SD = 4.96), and social scores (M = 18.0, SD = 2.06).

**Table 15.** Descriptive statistics for Roots total and subscale scores by latent profile.

Profiles	*n*	(%)	Roots total M (SD)	Relationship with the family M (SD)	Relationships with the self M (SD)	Social Relationships M (SD)
1	749	(35.6%)	61.0 (10.3)	28.6 (5.51)	19.1 (5.76)	13.4 (3.13)
2	474	(22.5%)	70.1 (7.52)	36.0 (3.83)	18.0 (3.92)	16.0 (2.32)
3	222	(10.6%)	75.0 (6.66)	39.0 (3.28)	24.1 (4.40)	11.9 (2.10)
4	658	(31.3%)	88.6 (8.07)	41.6 (3.80)	29.1 (4.96)	18.0 (2.06)

Note: Scores are the sum of scores of the corresponding items.

#### 3.5.2. Comparison between LPA profiles and k-means clusters

**Figure 6.**
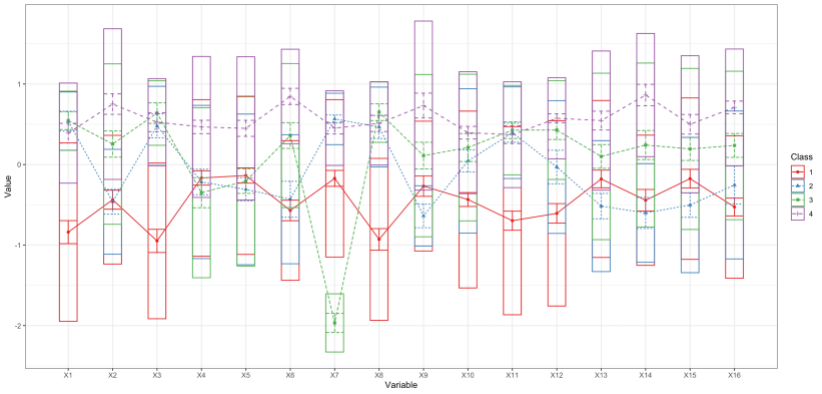
Latent profiler plot.

To reconcile the two classification approaches, we examined the overlap between the previously derived k-means solution (k = 2; hard partition) and the latent profile analysis (LPA; 4 probabilistic profiles, assigned using modal posterior membership) (Figure 6). Cross-tabulation of k-means cluster membership with LPA profile membership showed a very strong association [Pearson's χ²(3) = 1232.6, p < 0.001; Cramer's V = 0.77], indicating that both methods recover a common underlying structure while operating at different levels of granularity.

Within k-means Cluster 1, most participants were assigned to LPA Profile 1 (60.0%) or Profile 2 (33.1%), with only small proportions in Profiles 3 (6.5%) and 4 (0.4%). Conversely, within Cluster 2, the majority of participants were assigned to LPA Profile 4 (64.9%), followed by Profile 3 (15.0%), with smaller proportions in Profiles 1 (9.0%) and 2 (11.0%). Column-wise proportions showed that LPA Profile 4 was almost entirely composed of participants from Cluster 2 (99.4%), whereas LPA Profiles 1 and 2 were predominantly drawn from Cluster 1 (87.9% and 76.6%, respectively), and LPA Profile 3 mainly from Cluster 2 (68.0%). Overall, the LPA solution is broadly consistent with the original two-cluster structure but provides a more differentiated and probabilistic classification, effectively splitting each k-means cluster into more homogeneous subgroups with distinct Roots profiles.

Age differed significantly across the four latent profiles [Kruskal–Wallis χ²(3) = 42.05, p < 0.001]. Participants in Profile 4 were, on average, the oldest group (M = 24.6, SD = 7.08; median = 22, IQR = 6), whereas Profiles 2 and 3 included the youngest participants (M = 22.2, SD = 3.39 and M = 22.3, SD = 4.64, respectively; both median = 21). Profile 1 showed intermediate values (M = 23.4, SD = 5.50; median = 22, IQR = 4). Post-hoc pairwise Wilcoxon tests with Holm correction indicated that Profiles 2 and 3 were significantly younger than Profile 1 (p = 0.009 and p = 0.001, respectively), and Profile 4 was significantly older than all other profiles (all p ≤ 0.017). No significant age difference emerged between Profiles 2 and 3 (p = 0.140).

Sociodemographic and academic characteristics differed substantially across the four latent profiles (Table 16). Sex showed small but significant differences across profiles [χ²(3) = 41.44, p < 0.001, V = 0.14], with Profile 3 presenting a relatively higher proportion of women than Profiles 1 and 2. Age category also varied significantly, although with a small effect size [χ²(6) = 51.22, p < 0.001, V = 0.11], with Profiles 1 and 4 containing a slightly higher proportion of older students.

Strong and highly significant differences were observed in variables reflecting students' religious orientation. Both religious faith and religious category showed systematic distributional shifts across profiles (all p < 0.001, V ≈ 0.10). These differences, although modest in effect size, indicate distinct value- and belief-oriented patterns across Roots profiles.

Academic variables demonstrated even more pronounced differentiation. Course of studies, area of study, level of study, and course year all differed significantly across profiles (all p < 0.001), with effect sizes ranging from small to moderate (V = 0.10–0.24). For example, Profile 3 included a concentrated group of students in specific tracks and more advanced levels of study, whereas Profiles 1 and 4 were more heterogeneous.

Geographical and contextual variables yielded some of the strongest effects. Students' city of study, and location were all strongly associated with profile membership (all p < 0.001; V ≈ 0.18–0.22). The most notable differences emerged for private university status, offsite status, and scholarship in a College of Merit, which displayed very large effect sizes (V = 0.49–0.63). In particular, Profile 3 was overwhelmingly composed of students from private institutions, offsite students, and those holding a merit-based scholarship.

Overall, the latent profiles were highly distinct in terms of sociodemographic, contextual, and academic characteristics, supporting the ecological validity of the four-profile solution.

**Table 16.** Sociodemographic and academic characteristics across the four latent profiles.

Variable	Category	Profile 1	Profile 2	Profile 3	Profile 4	χ²(df)	p	V
Sex	0	150 (20.0%)	80 (16.9%)	69 (31.1%)	201 (30.5%)	41.44 (3)	5.3e-09	0.140
	1	599 (80.0%)	394 (83.1%)	153 (68.9%)	457 (69.5%)			
Age category	0	562 (75.0%)	397 (83.8%)	188 (84.7%)	461 (70.1%)	51.22 (6)	2.7e-09	0.110
	1	165 (22.0%)	76 (16.0%)	29 (13.1%)	161 (24.5%)			
	2	22 (2.9%)	1 (0.2%)	5 (2.3%)	36 (5.5%)			
Religious faith	0	322 (43.0%)	249 (52.5%)	136 (61.3%)	396 (60.2%)	74.10 (27)	2.9e-06	0.108
	1	22 (2.9%)	6 (1.3%)	3 (1.4%)	9 (1.4%)			
	2	13 (1.7%)	11 (2.3%)	4 (1.8%)	13 (2.0%)			
	4	7 (0.9%)	4 (0.8%)	2 (0.9%)	4 (0.6%)			
	5	1 (0.1%)	1 (0.2%)	0 (0.0%)	4 (0.6%)			
	6	1 (0.1%)	1 (0.2%)	0 (0.0%)	1 (0.2%)			
	7	15 (2.0%)	4 (0.8%)	2 (0.9%)	6 (0.9%)			
	8	252 (33.6%)	141 (29.7%)	44 (19.8%)	134 (20.4%)			
	9	116 (15.5%)	56 (11.8%)	31 (14.0%)	89 (13.5%)			
	3	0 (0.0%)	1 (0.2%)	0 (0.0%)	2 (0.3%)			
Religious category	0	322 (43.0%)	249 (52.5%)	136 (61.3%)	396 (60.2%)	58.42 (9)	2.7e-09	0.096
	1	59 (7.9%)	28 (5.9%)	11 (5.0%)	39 (5.9%)			
	2	252 (33.6%)	141 (29.7%)	44 (19.8%)	134 (20.4%)			
	3	116 (15.5%)	56 (11.8%)	31 (14.0%)	89 (13.5%)			
Course of studies	0	361 (48.2%)	274 (57.8%)	71 (32.0%)	413 (62.8%)	369.44 (27)	8.0e-62	0.242
	1	77 (10.3%)	22 (4.6%)	77 (34.7%)	29 (4.4%)			
	2	20 (2.7%)	6 (1.3%)	22 (9.9%)	2 (0.3%)			
	3	51 (6.8%)	30 (6.3%)	0 (0.0%)	41 (6.2%)			
	4	60 (8.0%)	41 (8.6%)	5 (2.3%)	51 (7.8%)			
	5	69 (9.2%)	39 (8.2%)	36 (16.2%)	31 (4.7%)			
	6	10 (1.3%)	4 (0.8%)	0 (0.0%)	20 (3.0%)			
	7	33 (4.4%)	26 (5.5%)	1 (0.5%)	24 (3.6%)			
	8	39 (5.2%)	13 (2.7%)	0 (0.0%)	18 (2.7%)			
	9	29 (3.9%)	19 (4.0%)	10 (4.5%)	29 (4.4%)			
Area of study	0	507 (67.7%)	322 (67.9%)	177 (79.7%)	471 (71.6%)	68.98 (12)	5.0e–10	0.105
	1	51 (6.8%)	30 (6.3%)	0 (0.0%)	41 (6.2%)			
	2	103 (13.8%)	71 (15.0%)	6 (2.7%)	95 (14.4%)			
	3	69 (9.2%)	39 (8.2%)	36 (16.2%)	31 (4.7%)			
	4	19 (2.5%)	12 (2.5%)	3 (1.4%)	20 (3.0%)			
Level of study	0	595 (79.4%)	400 (84.4%)	114 (51.4%)	544 (82.7%)	114.25 (3)	1.3e–24	0.233
	1	154 (20.6%)	74 (15.6%)	108 (48.6%)	114 (17.3%)			
Course year	1	174 (23.2%)	99 (20.9%)	78 (35.1%)	137 (20.8%)	67.22 (15)	1.4e–08	0.103
	2	291 (38.9%)	222 (46.8%)	51 (23.0%)	317 (48.2%)			
	3	146 (19.5%)	84 (17.7%)	42 (18.9%)	124 (18.8%)			
	4	47 (6.3%)	20 (4.2%)	19 (8.6%)	34 (5.2%)			
	5	64 (8.5%)	29 (6.1%)	22 (9.9%)	32 (4.9%)			
	6	27 (3.6%)	20 (4.2%)	10 (4.5%)	14 (2.1%)			
City where studying	0	444 (59.3%)	229 (48.3%)	206 (92.8%)	278 (42.2%)	214.35 (24)	1.7e–32	0.184
	1	34 (4.5%)	30 (6.3%)	2 (0.9%)	48 (7.3%)			
	2	9 (1.2%)	10 (2.1%)	1 (0.5%)	13 (2.0%)			
	3	125 (16.7%)	91 (19.2%)	8 (3.6%)	106 (16.1%)			
	4	121 (16.2%)	89 (18.8%)	5 (2.3%)	182 (27.7%)			
	6	4 (0.5%)	9 (1.9%)	0 (0.0%)	10 (1.5%)			
	7	9 (1.2%)	15 (3.2%)	0 (0.0%)	17 (2.6%)			
	8	3 (0.4%)	0 (0.0%)	0 (0.0%)	2 (0.3%)			
	5	0 (0.0%)	1 (0.2%)	0 (0.0%)	2 (0.3%)			
Location	0	125 (16.7%)	91 (19.2%)	8 (3.6%)	106 (16.1%)	201.91 (9)	1.3e–38	0.179
	1	444 (59.3%)	229 (48.3%)	206 (92.8%)	278 (42.2%)			
	2	46 (6.1%)	41 (8.6%)	3 (1.4%)	66 (10.0%)			
	3	134 (17.9%)	113 (23.8%)	5 (2.3%)	208 (31.6%)			
Private University	0	627 (83.7%)	453 (95.6%)	26 (11.7%)	611 (92.9%)	843.17 (3)	1.9e–182	0.633
	1	122 (16.3%)	21 (4.4%)	196 (88.3%)	47 (7.1%)			
Offsite	NA	109 (14.6%)	6 (1.3%)	192 (86.5%)	12 (1.8%)	1051.80 (6)	5.6e–224	0.500
	0	433 (57.8%)	307 (64.8%)	22 (9.9%)	383 (58.2%)			
	1	207 (27.6%)	161 (34.0%)	8 (3.6%)	263 (40.0%)			
Scholarship (college of merit)	NA	114 (15.2%)	6 (1.3%)	191 (86.0%)	17 (2.6%)	1004.13 (6)	1.1e–213	0.489
	0	529 (70.6%)	384 (81.0%)	21 (9.5%)	497 (75.5%)			
	1	106 (14.2%)	84 (17.7%)	10 (4.5%)	144 (21.9%)			

Note: Sex: 0 = Male, 1 = Female. Age category: 0 = Young, 1 = Young adults, 2 = Middle-aged. Religious faith: 0 = Catholic, 1= Orthodox, 2 = Other Christian denomination, 3 =Jewish, 4 = Muslim, 5 = Buddhist, 6 = Hindu, 7 = Other religion, 8 = Atheist/Agnostic, 9 = Indifferent; Religious category: 0 = Catholic, 1 = Other religion, 2 = Atheist/Agnostic, 3 = Indifferent. Course of studies: 0 = Nursing sciences, 1 = Medicine and dentistry, 2 = Nutrition, 3 = Science (Mathematics, Physics, Chemistry, Biology, Biotechnology etc.), 4 = Humanities (philosophy, literature, communication, law etc.), 5 = Engineering, 7 = Educational sciences, 8 = Economy, 9 = Psychology, 10 = Others. Area of study: 0 = Health science, 1 = Science, 2 = Humanities, 3 = Engineering, 4 = Others. Level of study: 0 = Bachelor's degree, 1 = Master's degree. City where studying: 0 = Roma, 1 = Bari, 2 = Caserta, 3 = Torino, 4 = Catania, 5 = Napoli, 6 = Sassari, 7 = Palermo, 8 = Lecce. Location: 0 = North, 1 = Centre, 2 = South, 3 = Main Islands. Private University, Offsite and Scholarship (college of merit): 0 = No, 1 = Yes.

## 4. Discussion

This study investigated the perceived origins of perfectionism in university students, specifically early relational experiences, self-acceptance, and social influences, using the Roots questionnaire, and examined how these aspects relate to students' sociodemographic and academic characteristics.

Overall, high ratings emerged for the dimension Relationships with the Family, suggesting the presence of emotional support, mutual trust, and a good degree of decision-making autonomy. These factors, extensively recognized in the literature as protective against perfectionistic traits [58], indicate that families play a central role in students' emotional development. The family environment often functions as a secure base, fostering self-efficacy and reducing reliance on external standards for self-worth [59]. These results align with theoretical models emphasizing the family's role in shaping self-concept and regulating personal expectations [60]. In cohesive families, particularly common in Italian culture, a sense of belonging may protect against socially prescribed perfectionism, in which approval is contingent on performance [61],[62]. Conversely, children who receive parental support contingent on achievement, even in emotionally positive contexts, tend to develop more unstable self-esteem dependent on external validation [63], a dynamic linked to maladaptive perfectionism, where acceptance is conditional on performance [64].

High scores at the Social Relationships dimension suggest that many students perceive themselves as accepted and recognized by others, with at least moderate interpersonal trust. This result is consistent with Mediterranean cultural patterns, where family cohesion and familism provide emotional support and buffer socio-relational stress, contributing to well-being during the transition to adulthood [65],[66]. These findings highlight that not all perfectionists experience social disconnection. For example, self-oriented perfectionism has been associated with social connectedness, whereas other-oriented and socially prescribed perfectionism are linked to hostility, social isolation, and reduced capacity for authentic relationships [67],[68]. In competitive contexts such as universities, where recognition is tied to academic performance and prestige, these dynamics are particularly relevant [67]. Moreover, low interpersonal trust and social withdrawal, especially among students with depressive symptoms, have been associated with academic burnout [69]. Interventions targeting self-compassion and socio-emotional skills, such as compassion-focused therapy, may mitigate self-criticism and social disconnection, thereby supporting psychosocial well-being [70].

In the dimension Relationships with the Self, more fragile patterns emerge. Although students report a moderate sense of self-esteem, they also disclose elevated fear of failure and feelings of unworthiness. These features, core to perfectionistic concerns, are strongly associated with anxiety, depression, suicidal ideation, eating disorders, and academic procrastination [11],[71]. Low levels of self-compassion, which are inversely related to maladaptive perfectionism, further underscore this vulnerability [19]. Thus, even when students show adaptive goal orientation, self-critical tendencies remain significant and potentially maladaptive.

Comparisons across sociodemographic groups revealed that younger students scored higher in Relationships with the Family, likely reflecting both continued proximity to the parental household and limited exposure to environments requiring full independence. Evidence suggests that family support during early adulthood buffers against unhealthy perfectionism [72]. At later ages, however, increased self-reliance and exposure to academic and role-related pressures may reduce perceived family support while intensifying perfectionistic concerns [73].

Gender differences also emerged. Women were more likely to report fear of failure and self-criticism, despite scoring higher in Relationships with the Self. This indicates that, although women perceived some self-efficacy, they also experienced stronger internal pressure and heightened sensitivity to errors. Male students, by contrast, reported higher perceived family support and external recognition, factors that may buffer the effect of the critical aspects of perfectionism. These findings align with studies showing that women more often exhibit perfectionistic concerns, characterized by failure-related anxiety, fear of judgment, and experiences of impostorism [73]–[75]. Gender, therefore, appears to shape how perfectionism is expressed, rather than determining its positivity or negativity outright.

Students from southern and insular regions reported greater perceived family support than those from northern regions. These findings reflect Mediterranean cultural traditions, particularly in southern Italy, where family relationships are marked by high emotional interdependence, solidarity, and centrality of the household [76],[77].

Catholic students, especially practicing individuals, also reported higher scores in Relationships with the Family (acceptance, trust, and autonomy). At the same time, religious practice was associated with greater fear of failure and feelings of inadequacy. While religiosity may foster cohesion and support, rigid internalization of norms can lead to self-criticism, guilt, and moral anxiety [78].

Health Sciences students, compared to Science/Engineering students, showed stronger indicators of family and self-related support. This may reflect professional socialization processes typical of health-related training, which emphasize care, empathy, and relational skills [79]. Nonetheless, health professions remain highly vulnerable to maladaptive perfectionism and burnout [80],[81]. Additionally, undergraduates reported higher self-confidence and lower fear of failure than master's students, likely due to the escalating demands of academic progression [82]. This suggests that personal and relational resources developed during training may be challenged by heightened expectations, highlighting the need for targeted preventive and supportive interventions.

Students receiving merit-based scholarships reported stronger internal pressure and a heightened need to maintain high standards. This reflects contingent self-worth, in which self-esteem depends on performance and external validation [83]. Although achievement motivation may increase, this dynamic also elevates maladaptive perfectionism and risks to psychological well-being [84].

Off-campus students generally reported more protective profiles in Relationships with the Self and Relationships with the Family, characterized by greater autonomy and confidence in personal abilities. Living independently appears to foster self-management skills and resilience [85]. Similarly, students at private universities displayed comparable protective indicators, despite potentially more competitive contexts. One explanation is that private institutions cultivate autonomy-supportive environments that encourage competence, choice, and belonging, factors known to mitigate perfectionistic concerns and enhance well-being [86]. Overall, these findings confirm that perfectionism in university students is not monolithic but shaped by socio-cultural, identity-related, and academic contexts. Family culture, gender roles, and educational contexts may function either as protective or risk factors, modulating both the expression and consequences of perfectionism.

Cluster analysis identified two distinct student profiles. Cluster 1 is marked by low perceptions of protective factors, particularly in self-esteem, family support, and social trust. These students report greater vulnerability to fear of failure and external validation, a pattern consistent with “mixed perfectionists” in the 2 × 2 model [87], who combine achievement striving with high rigidity and self-criticism. Such profiles are linked to higher stress, anxiety, and burnout [30],[88]. Cluster 2, by contrast, exhibits stronger family support, social acceptance, trust, and self-esteem, coupled with lower fear of failure, characteristics of adaptive perfectionism, where motivation for excellence is balanced by error tolerance and intrinsic self-worth [16]. This profile has been associated with emotional regulation and higher academic satisfaction [84],[89].

These profiles carry practical implications. Cluster 1 represents a vulnerable subgroup that could benefit from interventions reducing self-criticism and strengthening adaptive coping, such as mindful self-compassion [90] or cognitive restructuring [91]. Cluster 2 may benefit from interventions focused on maintaining motivation while preserving flexibility and resilience, such as programs emphasizing constructive error management [92]. The cluster analysis confirms international evidence that perfectionism in students manifests in qualitatively distinct forms [93]. Recognizing these profiles enables the design of tailored interventions that sustain academic achievement without compromising mental health.

The LPA refined and expanded the picture obtained from the cluster analysis. While k-means identified only two groups with modest separation, LPA supported a four-profile structure with high classification accuracy (entropy = 0.93). This indicates that beyond a simple low- vs. high-protection split on Roots, students show multiple qualitatively distinct patterns of family, personal, and social resources. The four profiles ranged from globally low protection (Profile 1) to uniformly high protection (Profile 4), with two mixed profiles capturing meaningful combinations of strengths and weaknesses (e.g., strong family support but weaker self-related protection; or strong family/self resources paired with weaker social relationships).

The methods showed strong concordance: LPA essentially subdivided each k-means cluster into more homogeneous subgroups, revealing important within-cluster heterogeneity relevant for understanding different etiological pathways of perfectionistic concerns. Methodologically, k-means provided a simple initial structure, whereas LPA offered greater precision and a richer, theory-aligned typology. Together, they suggest that combining both methods can be valuable when analyzing complex psychosocial datasets.

Implications for practice and research follow from the profile-specific patterns. Students in Profile 1 (globally low protection) appear most vulnerable and may benefit from interventions targeting self-criticism and fear of failure alongside family and social skill-building. Profile 2 highlights a subgroup with intact family resources but lower self-related protection; interventions focused on self-compassion, adaptive error appraisal, and decoupling self-worth from performance may be particularly beneficial. Profile 3 underscores the relevance of social connectedness and trust as distinct targets even when resources from family and self are strong (e.g., peer-based and group interventions to enhance belonging). Profile 4, while most protected, may profit from maintenance-oriented support that preserves flexibility and resilience under escalating academic demands. Future work should test whether these profiles differentially predict academic burnout, distress, and performance trajectories, and whether tailored interventions yield profile-specific benefits.

### 4.1. Strengths and limitations

Strengths of this study include the focus on the understudied etiological factors of perfectionism using Roots, a valid questionnaire tailored to the Italian university population, with a large (n = 2103) and geographically diverse sample. The statistical analyses conducted, particularly profiling analyses, provide a nuanced and robust understanding of the profile structure of the familiar, personal, or social origin of perfectionism and its relationships with sociodemographic variables.

However, several limitations should be acknowledged. The use of convenience sampling introduces potential selection bias, and the sample cannot be considered representative of the entire Italian university student population. Moreover, the cross-sectional design prevents causal inference, and reliance on self-report data may be subject to social desirability and recall bias.

From a psychometric standpoint, the inspection of item–total correlations and bifactor indices suggests that the Roots is largely driven by a general relational–self evaluative factor rather than by three equally strong and independent dimensions. Most Family and Self items showed adequate item–total correlations, whereas the Social Relationships items displayed consistently low values, in line with the weaker internal consistency of this subscale. In this context, the total Roots score emerges as the most robust indicator of the underlying construct, and the three subscales should be interpreted with some caution, particularly Social Relationships. Consistent with this evidence, all person-centered analyses (k-means clustering and latent profile analysis) were conducted on the full set of n = 16 items rather than on subscale scores, thereby capitalizing on the item-level information and on the predominance of the general factor when identifying student profiles.

Future research adopting longitudinal and mixed methods designs should evaluate the impact of interventions aimed at reinforcing the adaptive aspects of perfectionism and reducing its maladaptive effects.

## 5. Conclusions

The origins of perfectionism in university students are closely linked with family dynamics, self-perceptions, and socio-cultural environments. While family support and relational resources function as protective factors, self-criticism and fear of failure remain significant vulnerabilities in competitive academic settings. The contrast between “mixed” and “adaptive” perfectionist profiles underlines the need for interventions that reduce the negative consequences of maladaptive perfectionism while reinforcing its functional elements, such as motivation and pursuit of excellence. Initiatives aimed at enhancing psychological well-being are essential to support students in their academic and personal development, preserving the adaptive aspects of perfectionism and mitigating its maladaptive outcomes.

## Use of AI tools declaration

The authors declare they have not used Artificial Intelligence (AI) tools in the creation of this article.


