# Supplementary material for: Measuring the origins of perfectionism with the Roots questionnaire in university students: sociodemographic and educational correlates
Source: AIMS Public Health. 2026 Mar 16;13(1):353–94. doi: 10.3934/publichealth.2026019 (PMC13084365; doi:10.3934/publichealth.2026019)
Supplement: Supplementary file 1 [file publichealth-13-01-019-s001.pdf]

Public Health

AIMS Public Health, 13(1): 353–394.
DOI: 10.3934/publichealth.2026019
Received: 29 October 2025
Revised: 05 January 2026
Accepted: 09 January 2026
Published: 16 March 2026

https://www.aimspress.com/journal/aimsph

Research article

Measuring the origins of perfectionism with the roots questionnaire in university students: sociodemographic and educational correlates

Elena Sandri[1,*], Agnese Broccolo[2], Anna Marchetti[3,4,*], Anna De Benedictis[3], Giorgia Petrucci[5], Laura Campanozzi[6], Mattia Bozzetti[7], Teresa Rea[8], Rosaria Alvaro[2], Maria Grazia De Marinis[3,4] and Michela Piredda[3]

1 Faculty of Medicine and Health Sciences, Catholic University of Valencia San Vicente Mártir c/Quevedo, 2–46001, Valencia, Spain
2 Department of Biomedicine and Prevention, University of Rome Tor Vergata, Via Montpellier, 1–00133, Rome, Italy
3 Department of Medicine and Surgery, Research Unit Nursing Science, Campus Bio-Medico di Roma University, Via Alvaro del Portillo 21–00128 Rome, Italy
4 Research Unit of Nursing Palliative Care, Fondazione Policlinico Universitario Campus Bio-Medico, Via Alvaro del Portillo, 200–00128, Rome, Italy
5 Research Unit of Orthopaedic and Trauma Surgery, Fondazione Policlinico Universitario Campus Bio-Medico, Via Alvaro del Portillo, 200–00128 Rome, Italy
6 Research Unit of Bioethics and Humanities, Campus Bio-Medico di Roma University, Via Alvaro del Portillo, 21–00128 Rome, Italy
7 Direction of Health Professions, ASST Cremona, 26100 Cremona, Italy
8 Department Public Health, Università degli Studi di Napoli Federico II, Via Pansini, 5–80131–Naples

* **Correspondence:** Elena Sandri: Emil: elena.sandri@ucv.es; Anna Marchetti: Email: anna.marchetti@policlinicocampus.it.

Table S1. STROBE Statement—Checklist of items that should be included in reports of *cross-sectional studies*.

	Item No	Recommendation
Title and abstract	1	(a) Indicate the study's design with a commonly used term in the title or the abstract. Page 1<br>(b) Provide in the abstract an informative and balanced summary of what was done and what was found. Page 2,3
Introduction		
Background/rationale	2	Explain the scientific background and rationale for the investigation being reported. Pages 2,3
Objectives	3	State specific objectives, including any prespecified hypotheses. Page 3
Methods		
Study design	4	Present key elements of study design early in the paper. Pages 3–10
Setting	5	Describe the setting, locations, and relevant dates, including periods of recruitment, exposure, follow-up, and data collection. Pages 10
Participants	6	(a) Give the eligibility criteria, and the sources and methods of selection of participants. Pages 10
Variables	7	Clearly define all outcomes, exposures, predictors, potential confounders, and effect modifiers. Give diagnostic criteria, if applicable. Pages 3–10
Data sources/ measurement	8*	For each variable of interest, give sources of data and details of methods of assessment (measurement). Describe comparability of assessment methods if there is more than one group. Pages 3–10
Bias	9	Describe any efforts to address potential sources of bias. Pages 3–10
Study size	10	Explain how the study size was arrived. Pages 3–10
Quantitative variables	11	Explain how quantitative variables were handled in the analyses. If applicable, describe which groupings were chosen and why. Pages 3–10
Statistical methods	12	(a) Describe all statistical methods, including those used to control for confounding. Pages 5–10<br>(b) Describe any methods used to examine subgroups and interactions. Pages 5–10<br>(c) Explain how missing data were addressed. Pages 5–10<br>(d) If applicable, describe analytical methods taking account of sampling strategy. Not applicable<br>(e) Describe any sensitivity analyses. Pages 5–10
Results		
Participants	13*	(a) Report numbers of individuals at each stage of study—eg numbers potentially eligible, examined for eligibility, confirmed eligible, included in the study, completing follow-up, and analysed. Pages 10–32<br>(b) Give reasons for non-participation at each stage. Page 10–32<br>(c) Consider use of a flow diagram. Not applicable
Descriptive data	14*	(a) Give characteristics of study participants (eg demographic, clinical, social) and information on exposures and potential confounders. Pages 10–12<br>(b) Indicate number of participants with missing data for each variable of interest. Pages 10–32
Outcome data	15*	Report numbers of outcome events or summary measures. Pages 10–32
Main results	16	(a) Give unadjusted estimates and, if applicable, confounder-adjusted estimates and their precision (eg, 95% confidence interval). Make clear which confounders were adjusted for and why they were included. Pages 10–32<br>(b) Report category boundaries when continuous variables were categorized. Pages 10–32<br>(c) If relevant, consider translating estimates of relative risk into absolute risk for a meaningful time period. Pages 10–32

Continued on next page

	Item No	Recommendation
Other analyses	17	Report other analyses done—eg analyses of subgroups and interactions, and sensitivity analyses. Pages 10–32
Discussion		
Key results	18	Summarise key results with reference to study objectives. Pages 32–35
Limitations	19	Discuss limitations of the study, taking into account sources of potential bias or imprecision. Discuss both direction and magnitude of any potential bias. Pages 35–36
Interpretation	20	Give a cautious overall interpretation of results considering objectives, limitations, multiplicity of analyses, results from similar studies, and other relevant evidence. Pages 32–36
Generalisability	21	Discuss the generalisability (external validity) of the study results. Pages 32–36
Other information		
Funding	22	Give the source of funding and the role of the funders for the present study and, if applicable, for the original study on which the present article is based. Not applicable

Note: *Give information separately for exposed and unexposed groups. An Explanation and Elaboration article discusses each checklist item and gives methodological background and published examples of transparent reporting. The STROBE checklist is best used in conjunction with this article (freely available on the Web sites of PLoS Medicine at http://www.plosmedicine.org/, Annals of Internal Medicine at http://www.annals.org/, and Epidemiology at http://www.epidem.com/). Information on the STROBE Initiative is available at www.strobe-statement.org.

AIMS Press

© 2026 the Author(s), licensee AIMS Press. This is an open access article distributed under the terms of the Creative Commons Attribution License (https://creativecommons.org/licenses/by/4.0)
